# nsP4 Is a Major Determinant of Alphavirus Replicase Activity and Template Selectivity

**DOI:** 10.1128/JVI.00355-21

**Published:** 2021-09-27

**Authors:** Laura Sandra Lello, Koen Bartholomeeusen, Sainan Wang, Sandra Coppens, Rennos Fragkoudis, Luke Alphey, Kevin K. Ariën, Andres Merits, Age Utt

**Affiliations:** a Institute of Technology, University of Tartu, Tartu, Estonia; b Department of Biomedical Sciences, Institute of Tropical Medicine, Antwerp, Belgium; c University of Nottingham, School of Veterinary Medicine and Science, Loughborough, United Kingdom; d The Pirbright Institute, Woking, United Kingdom; e Department of Biomedical Sciences, University of Antwerp, Antwerp, Belgium; University of North Carolina at Chapel Hill

**Keywords:** RNA polymerases, RNA replication, alphavirus, genetic recombination, replicase

## Abstract

Alphaviruses have positive-strand RNA genomes containing two open reading frames (ORFs). The first ORF encodes the nonstructural (ns) polyproteins P123 and P1234 that act as precursors for the subunits of the viral RNA replicase (nsP1 to nsP4). Processing of P1234 leads to the formation of a negative-strand replicase consisting of nsP4 (RNA polymerase) and P123 components. Subsequent processing of P123 results in a positive-strand replicase. The second ORF encoding the structural proteins is expressed via the synthesis of a subgenomic RNA. Alphavirus replicase is capable of using template RNAs that contain essential *cis*-active sequences. Here, we demonstrate that the replicases of nine alphaviruses, expressed in the form of separate P123 and nsP4 components, are active. Their activity depends on the abundance of nsP4. The match of nsP4 to its template strongly influences efficient subgenomic RNA synthesis. nsP4 of Barmah Forest virus (BFV) formed a functional replicase only with matching P123, while nsP4s of other alphaviruses were compatible also with several heterologous P123s. The P123 components of Venezuelan equine encephalitis virus and Sindbis virus (SINV) required matching nsP4s, while P123 of other viruses could form active replicases with different nsP4s. Chimeras of Semliki Forest virus, harboring the nsP4 of chikungunya virus, Ross River virus, BFV, or SINV were viable. In contrast, chimeras of SINV, harboring an nsP4 from different alphaviruses, exhibited a temperature-sensitive phenotype. These findings highlight the possibility for formation of new alphaviruses via recombination events and provide a novel approach for the development of attenuated chimeric viruses for vaccination strategies.

**IMPORTANCE** A key element of every virus with an RNA genome is the RNA replicase. Understanding the principles of RNA replicase formation and functioning is therefore crucial for understanding and responding to the emergence of new viruses. Reconstruction of the replicases of nine alphaviruses from nsP4 and P123 polyproteins revealed that the nsP4 of the majority of alphaviruses, including the mosquito-specific Eilat virus, could form a functional replicase with P123 originating from a different virus, and the corresponding chimeric viruses were replication-competent. nsP4 also had an evident role in determining the template RNA preference and the efficiency of RNA synthesis. The revealed broad picture of the compatibility of the replicase components of alphaviruses is important for understanding the formation and functioning of the alphavirus RNA replicase and highlights the possibilities for recombination between different alphavirus species.

## INTRODUCTION

Alphaviruses (family *Togaviridae*) are positive-strand RNA viruses. They have an RNA genome of approximately 12 kb in length, with a 5′ cap structure and a 3′ poly(A) tail, and contain two open reading frames (ORF). The majority of alphaviruses known to date are arboviruses, i.e., they are transmitted between vertebrate hosts by arthropod vectors, predominantly mosquitoes (1). In addition, a number of insect-specific viruses (ISV), belonging to the *Alphavirus* genus, have been recently discovered (2–6). Alphaviruses infecting aquatic species are capable of replicating in mosquito cells (7), but their natural arthropod vector(s), if any, are not known. Arbovirus members of the genus *Alphavirus* are divided into several complexes forming three major clades (1). The Venezuelan equine encephalitis complex includes Venezuelan equine encephalitis virus (VEEV), the western equine encephalitis complex includes western equine encephalitis virus (WEEV) as well as Sindbis virus (SINV, the type member of the genus), and the Semliki Forest complex includes Semliki Forest virus (SFV), chikungunya virus (CHIKV), o’nyong-nyong virus (ONNV), mayaro virus (MAYV), and Ross River virus (RRV). Barmah Forest virus (BFV) is the only known member of the Barmah Forest complex. Other recognized complexes are the eastern equine encephalitis complex, Middelburg complex, and Ndumu complex (1). SFV and SINV serve as model viruses and have been extensively studied over the decades. Recently, more attention has been dedicated to the studies of alphaviruses associated with important human diseases, including CHIKV, ONNV, RRV and VEEV. These studies have revealed significant differences between alphaviruses belonging to different clades and complexes (8–11). Even viruses belonging to the same complex (12–15; see companion article [16]), including very closely related alphavirus species such as CHIKV and ONNV, often have significantly different biological properties (11, 17). These findings highlight the need to study each alphavirus individually. At the same time, there is a need to obtain additional general information, applicable to many or all alphaviruses, that can be used for understanding the basics of alphavirus molecular biology and evolution as well as for the development of universal antialphavirus strategies.

Mechanisms of replication complex (RC) formation and the RNA replication process are among the most conserved molecular features of alphaviruses. The first ORF of the alphavirus genome encodes four nonstructural (ns) proteins (nsP1 to 4) that are subunits of the RC. For most alphaviruses, they are produced in the form of polyprotein precursors P1234 or P123, with P1234 being synthesized by a readthrough of an in-frame opal termination codon between the nsP3 and nsP4 regions (18). The polyproteins are essential for the correct interactions between different components of the RCs as well as for their correct subcellular localization (19). The formation of polyprotein processing intermediates and mature nsPs via protease activity of the nsP2 region is therefore tightly regulated. Only one processing pathway has been shown to lead to the formation of functional RCs. The negative-strand replicase (P123+nsP4, also termed the early replicase) is formed by the cleavage of P1234 in a processing site located between the nsP3 and nsP4 regions (3/4 site) (20). The relative stability of P123 is needed in order to allow the transport of the replicase precursors to the plasma membrane, where the formation of membrane-bound RC (spherules) takes place (21). The interaction of P123 with plasma membrane is mediated by motifs located in the nsP1 region that acts as the membrane anchor of the RC. In an active form, nsP1 is assembled in a monotopic membrane-associated dodecameric ring which is important in RC assembly and controlling access to the inner side of RC (22, 23). Palmitoylation of cysteine residue(s) in the C-terminal part of nsP1 is used to tighten the association with membranes (24). This modification has different implications for different alphaviruses; for CHIKV, mutations preventing nsP1 palmitoylation are lethal, for SFV, the same mutations attenuate virus replication and cause accumulation of second site adaptive mutations, while for SINV, the effect of analogous mutation is relatively mild (14, 25, 26).

The release of nsP4 is the only cleavage of P1234 that is absolutely required for the activation of alphavirus RNA replication (27). In principle, a replicase consisting of P123 and nsP4 components is capable of synthesis of all RNAs required for alphavirus infection: in addition to the negative strand, new positive-strand genomic and subgenomic (SG) RNAs, used for expression of structural proteins, can be synthesized (28). This indicates that the alphavirus replicase consists of two relatively independent functional modules, nsP4 and P123. nsP4 is the RNA polymerase subunit of the replicase. On its own it has low solubility and minimal RNA polymerase activity (29, 30). Polymerase activity of nsP4 is stimulated by the presence of P123 and fragments thereof created by further processing. Several of these subunits participate in viral RNA synthesis and/or modification: nsP2 has an RNA helicase activity and together with nsP1 provides functions needed for RNA capping (22, 31–34). Acceleration of P123 processing often results in an attenuated phenotype and may even be lethal for a virus; such attenuating effects are usually more pronounced in mosquito cells than in mammalian cells (35–37, 75; see companion article [16]). Cleavage of the 1/2 site in P123 resulting in nsP1 and P23 is therefore a delayed and precisely timed event. It is rapidly followed by the *trans*-cleavage of P23 into nsP2 and nsP3, which together with nsP1 and nsP4, form the positive-strand replicase (nsP1-4; also termed the late replicase), which is responsible for the synthesis of large amounts of new genomic as well as SG RNAs (20).

Formation of a functional RC is a multistep process that depends on precise interactions between multiple viral (RNAs, nsPs and their precursors) and host components, including cell membranes and cellular proteins. These interactions have been studied extensively (11, 12, 22, 38–41), but thus far remain incompletely understood. In part, this is because the purification of functional RCs from alphavirus-infected cells or reconstitution of alphavirus replication systems in a test tube have been only moderately successful (42–45). The compatibility of ns-proteins of different alphaviruses is poorly understood, because chimeric viruses are not always functional and stringent biosafety measures apply to the highly pathogenic members of the genus alphavirus. It has, however, been shown that even for the closely related CHIKV and ONNV, only some, but not all, chimeric viruses are viable (17); the same has been observed for SFV and CHIKV containing swaps in the region corresponding to the C-terminal hypervariable domain of nsP3 (15). SFV chimeras, where nsP2 or its protease domain were replaced with their counterparts from SINV, were not viable (see companion article [16]). Similarly, replacing the nsPs of SINV with their counterparts from Eilat virus (EILV) resulted in nonviable chimeras (46). Thus, the use of homologous protein swapping in the context of the entire viral genome to study functional interactions between alphavirus nsPs has significant limitations. This hampers analysis of compatibility of the replicase components of closely and distantly related alphaviruses. Such information is, however, important for understanding the basic properties of (alpha)virus infection, including the RC formation. Furthermore, molecular insights in the compatibility of the replicase components are essential for understanding alphavirus evolution, where recombination between different viruses has been shown to represent an important mechanism (47).

Alphavirus *trans*-replicases allow the uncoupling of the ns-protein synthesis from its mRNA replication and eliminate the impact of potential adaptive mutations bound to arise during viral replication. Recently, the system has been used to analyze the dependence of alphavirus RNA synthesis on crucial cellular proteins and for analysis of the cross-utilization of the template RNAs by replicases of different alphaviruses (11, 13, 26). Here, we demonstrate that the replicases of SINV, CHIKV, ONNV, BFV, RRV, SFV, MAYV, VEEV, and EILV, expressed in the form of separate P123 and nsP4 components, have activities that are comparable to replicases expressed via a single P1234 precursor. The efficiency of viral RNA replication was found to be dependent on the relative abundance of the nsP4 component. Prevention of the processing of the P123 component reduced the activity of reconstituted replicases, and replicases reconstituted from separately expressed nsP1, noncleavable P23, and nsP4 had even lower activities. Using the promiscuous RNA template of SINV, it was found that nsP4 of BFV was capable of functioning only with matching P123, while nsP4 proteins from other viruses also formed active replicases with P123 belonging to heterologous viruses. In general, the P123 component of viruses belonging to the SFV complex was capable of forming highly active replicases with nsP4 originating from alphaviruses belonging to different complexes. Chimeras of SFV harboring the nsP4 of CHIKV, BFV, RRV, or SINV were viable. In contrast, the P123 components of VEEV and SINV were highly selective, the formation of a functional replicase was observed only with nsP4 belonging to the same virus, and chimeras of SINV, harboring the nsP4 of CHIKV, ONNV, BFV, RRV, SFV, MAYV, VEEV, or EILV, were not viable at 37°C. All these chimeric viruses did, however, replicate at 28°C. Using heterologous combinations of P123 and nsP4 from viruses belonging to the SFV complex, it was observed that combinations where the nsP4 and template RNA originated from the same virus had higher RNA synthesis activities and that the nsP4 component has an important role in the recognition/efficient use of the SG promoter. Revealing the broad picture of the compatibility of replicase subunits and template RNAs significantly contributes to our understanding of alphavirus RNA replicase formation and functioning and further highlights the possibilities of recombination between different alphavirus species. Finally, the approaches described here open new possibilities for the genetic attenuation of alphaviruses.

## RESULTS

**FIG 1:**
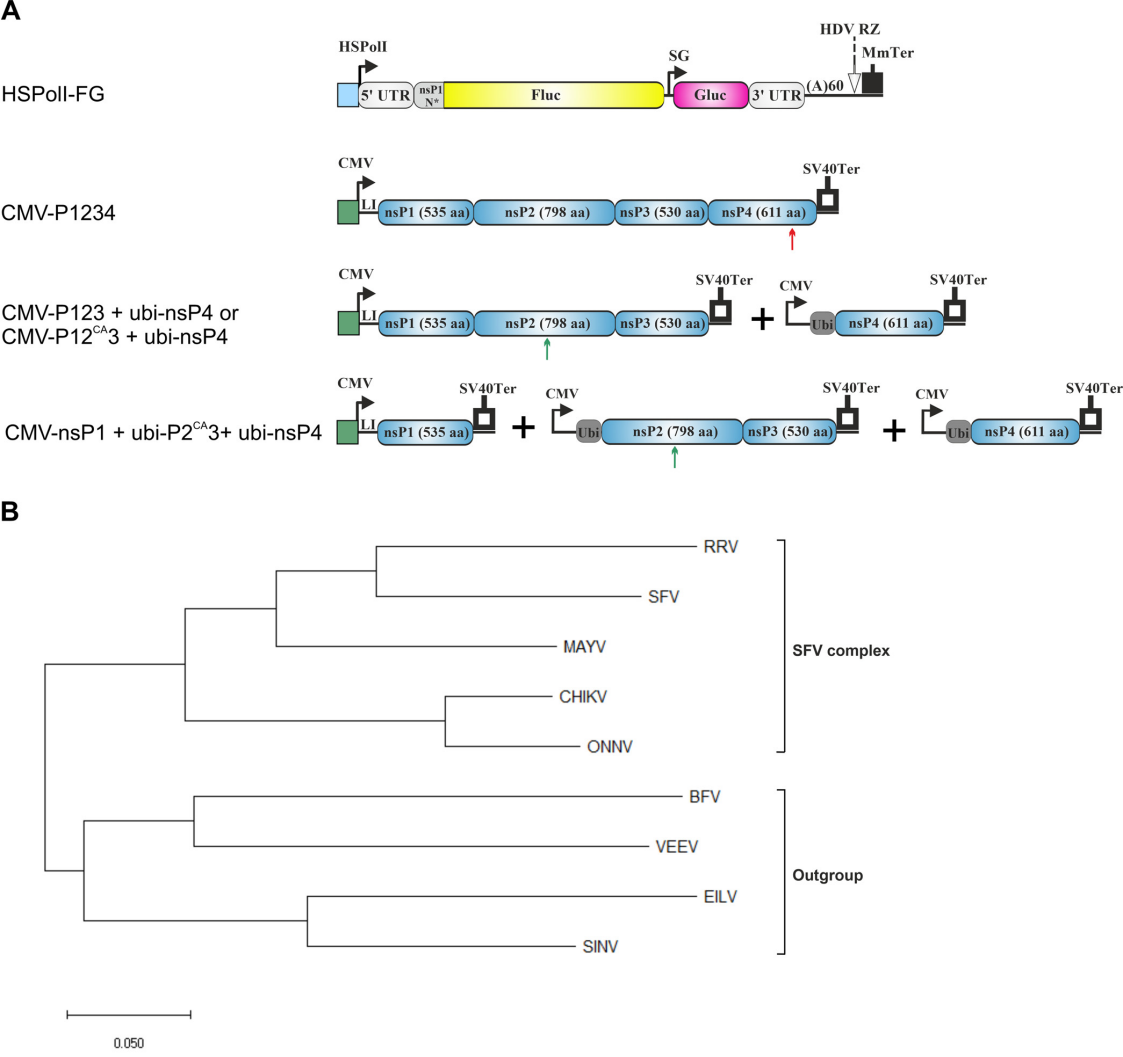
Expression plasmids for single-, two-, and three-component replicases. (A) Schematic presentation of template RNA (top) and replicase protein expression constructs. HSPolI, a truncated promoter (residues −211 to –1) for human RNA polymerase I; 5′ UTR, full-length 5′ UTR of an alphavirus; nsP1 N*, region encoding the N-terminal 77 to 114 amino acid residues of nsP1, depending on the virus; SG, SG promoter spanning (with respect to the termination codon of nsP4) from position –79 to the end of the intergenic region; 3′ UTR, truncated (last 110 residues) 3′ UTR of an alphavirus; HDV RZ, antisense strand ribozyme of hepatitis delta virus; MmTer, a terminator for RNA polymerase I in mice; CMV, immediate early promoter of human cytomegalovirus; LI, leader sequence of the herpes simplex virus thymidine kinase gene with artificial intron; SV40Ter, simian virus 40 late polyadenylation region; Ubi, sequence encoding human ubiquitin. The red arrow indicates the location of the GDD motif in nsP4; in polymerase-negative constructs, this was replaced by GAA. The green arrow indicates the location of the catalytic Cys residue in the active site of nsP2 protease; in protease-negative constructs, this was replaced by Ala residue. The vector backbones are not shown, and drawings are not to scale. (B) Phylogenetic tree of nsP4 RNA-dependent-RNA-polymerase proteins of the analyzed alphaviruses. The phylogenetic tree was constructed using evolutionary analysis with the maximum likelihood method and JTT matrix-based model. The tree is drawn to scale, with branch lengths measured in the number of substitutions per site. Evolutionary analysis was conducted using MEGA-X software.

The alphavirus *trans-*replicase system (Fig. 1A) allows experiments to be conducted in the absence of reversions, pseudoreversions, and compensatory changes (26). We and others have previously demonstrated that these systems are highly efficient tools to study the RC formation, the functional analysis of ns-proteins, and the requirements of host factors and functional interactions between ns-proteins and template RNA (11, 13, 48–50). The *trans-*replication system also allows the study of the compatibility of components of RNA replicases belonging to different alphaviruses without the need for chimeric virus construction, simplifying the analysis and circumventing biosafety concerns associated with chimeric viruses (11).

The ability of separately expressed P123 and nsP4 of SINV to form functional RNA replicase was demonstrated in the early 1990s and used to reveal the pathway leading to the alphavirus RNA replicase formation and maturation (20, 51, 52). A similar property has also been described for P123 and nsP4 of SFV and used to demonstrate that their expression triggers the formation of spherules, physical structures of alphavirus RCs, that are similar to these formed in virus-infected cells (49). Thus, the combinations of P123 and nsP4 components represent relevant tools for studies of the alphavirus RNA replicase. Here, this approach was applied to study the functional compatibility of two main components of the RNA replicase, the P123 (and cleavage products thereof) and nsP4 of alphaviruses belonging to different complexes.

Plasmids for the expression of P123 were constructed for five alphaviruses belonging to the SFV complex (SFV, CHIKV, ONNV, RRV, and MAYV) as well as for SINV, BFV, VEEV, and EILV, belonging to different complexes and here collectively referred to as “outgroup” alphaviruses (Fig. 1B). Similar to previous studies, in the nsP4 expression constructs, a sequence encoding ubiquitin was fused to the region encoding the N terminus of nsP4 (Fig. 1A); the removal of ubiquitin by cellular deubiquitinating enzymes results in an nsP4 with a native N-terminal Tyr-residue, which is critical for its activity (53). Plasmids expressing the template RNAs for all selected viruses have been previously described (11) and contain sequences encoding two reporters—firefly luciferase (Fluc) under the genomic promoter and *Gaussia* luciferase (Gluc) under the SG promoter (Fig. 1A).

For simplicity, here, the full-length RNA serving as the template for Fluc expression is termed “genomic RNA” (and its synthesis, “replication”), the RNA synthesized from the SG promoter and serving as the template for Gluc expression is termed “SG RNA” (and its synthesis, “transcription”), and all RNAs synthesized by *trans*-replicases are referred to as “viral RNAs.” The levels of Fluc and Gluc expression in cells, transfected with plasmids expressing the template RNA and corresponding polymerase negative-control replicase (P1234^GAA^), are similar for *trans-*replicases derived from different viruses. Therefore, the efficiency of replication and transcription was estimated by fold changes (“boost”) of corresponding reporter expression, as previously described (11). The replicase expressed in the form of P1234 precursor is here designated the “single-component replicase,” the P123 (or P12^CA^3)+nsP4 derived replicase is designated the “two-component replicase,” and the nsP1+P2^CA^3+nsP4 derived replicase is designated the “three-component replicase” (Fig. 1A). Combinations of plasmids expressing proteins and template RNAs from one and the same alphavirus are referred to as “matching combinations,” while the combinations expressing components from different viruses are referred to as “heterologous combinations.”

### Matching combinations of P123 and nsP4 form a highly active RNA replicase.

**FIG 2:**
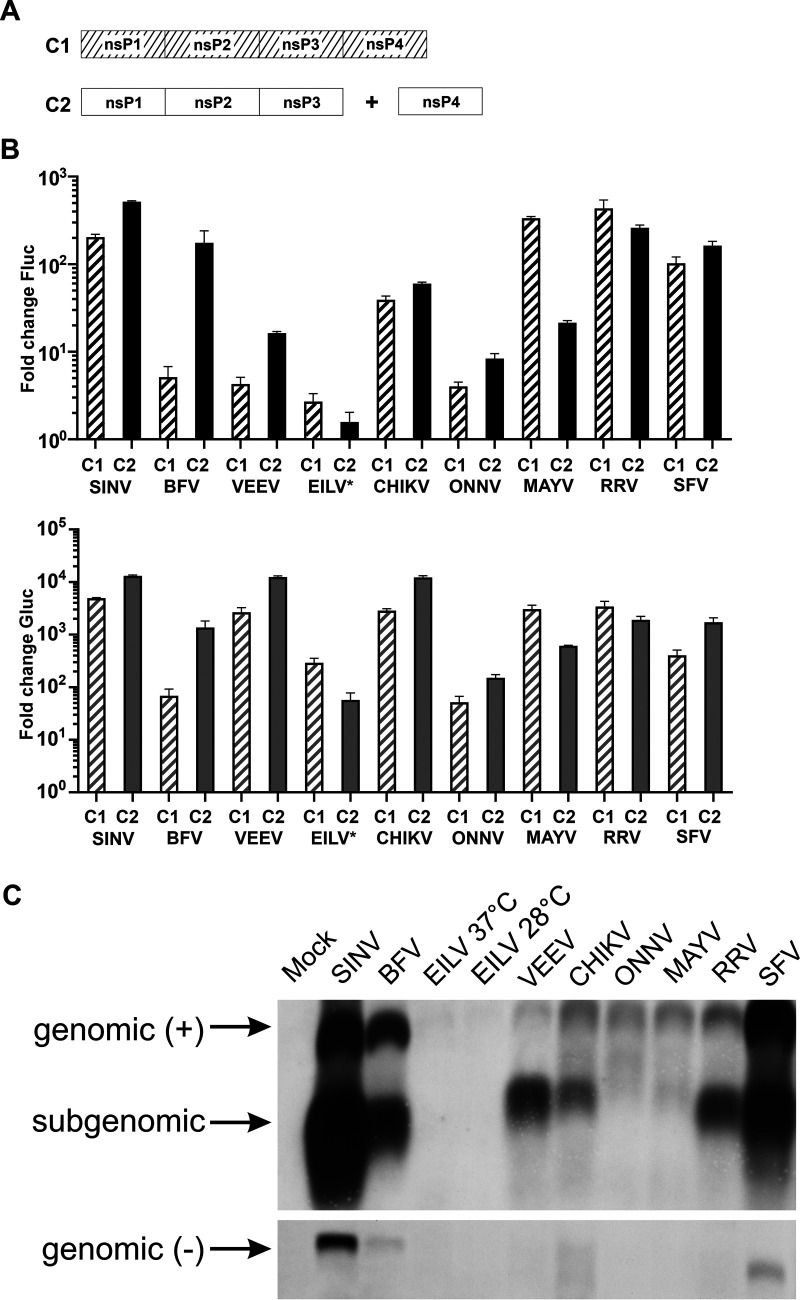
Single- and two-component replicases of alphaviruses have similar activities. (A). Schematic presentation of expressed replicase precursors. C1, single-component replicase from plasmids CMV-P1234-SINV and so on; C2, two-component replicase from plasmids CMV-P123-SINV+ CMV-ubi-nsP4-SINV and so on. (B) HEK293T cells in 96-well plates were cotransfected with matching pairs of CMV-P1234 and HSPolI-FG plasmids (C1) or with matching combinations of CMV-P123, CMV-ubi-nsP4 (in a 1:1 molar ratio), and HSPolI-FG plasmids (C2). As the negative control, CMV-P1234^GAA^, which encodes polyprotein lacking RNA polymerase activity, was used instead of CMV-P1234. Cells were incubated at 37°C and lysed for 18 h p.t.; cells transfected with plasmids containing sequences from EILV were incubated at 28°C and lysed for 48 h p.t. Fluc (marker of replication) and Gluc (marker of transcription) activities produced by active replicases were normalized to the P1234^GAA^ controls. The value obtained for the P1234^GAA^ controls was taken as 1. The means ± standard deviation (SD) of three independent experiments are shown. (C) HEK293T cells in 12-well plates were cotransfected with matching combinations of CMV-P123, CMV-ubi-nsP4 (in 1:1 molar ratio), and HSPolI-FG plasmids; control cells were mock-transfected. Cells were incubated as described for panel B, after which total RNA was extracted and analyzed by Northern blotting. Full-length “genomic” template RNA of positive (+) and negative (–) polarity and subgenomic RNA are indicated. Note that transcripts made by human RNA polymerase I using HSPolI-FG plasmids as templates comigrate with replicase-generated positive-strand genomic RNA and are detected by the same probe. The experiment was repeated twice with similar results; data from one experiment is shown.

To compare the activity of single- and two-component replicases, HEK293T cells were transfected using matching pairs of HSPolI-FG template expression plasmid and CMV-P1234 or matching combinations of HSPolI-FG and CMV-P123 supplemented with equimolar amounts of CMV-ubi-nsP4. It was observed that all two-component replicases were able to boost Fluc and Gluc expression. Furthermore, with the exceptions of EILV, MAYV, and RRV, the abilities of two-component replicases to synthesize viral RNAs exceeded those of single-component replicases (Fig. 2B and C), each of which (except EILV) has been previously demonstrated to produce very robust marker expression in human cells (11). In order to confirm that the observed boost of reporter activities does indeed reflect the synthesis of viral RNAs, a Northern blot analysis was performed. The amounts of all viral RNAs of EILV were below the level of detection, a finding that correlates with very low boosts of Fluc and Gluc expression by the EILV two-component replicase (compare Fig. 2B and C). Synthesis of positive-strand genomic RNAs by two-component replicases of VEEV, ONNV, and MAYV occurred at a low level; again, this is consistent with the moderate boosts of Fluc activity (compare Fig. 2B and C). Synthesis of negative-strand RNAs was below the detection limit for two-component replicases of ONNV, MAYV, VEEV, and RRV (Fig. 2C). Overall, the synthesis of positive-strand genomic RNAs and SG RNAs by all two-component replicases was extremely similar to that previously observed for single-component replicases of corresponding viruses (11). The only exception from this was BFV, for which the single-component replicase demonstrated only a modest ability of positive-strand RNA synthesis (11), while the two-component replicase was highly active (Fig. 2B and C). This correlates with the finding that for BFV the two-component replicase was significantly more active than single-component replicase for boosting both the Fluc (approximately 35-fold, *P* < 0.01) and Gluc (approximately 20-fold, *P* < 0.01) expression (Fig. 2B).

### Increasing the ratio of nsP4 to P123 expression plasmids increases RNA replication in transfected cells.

**FIG 3:**
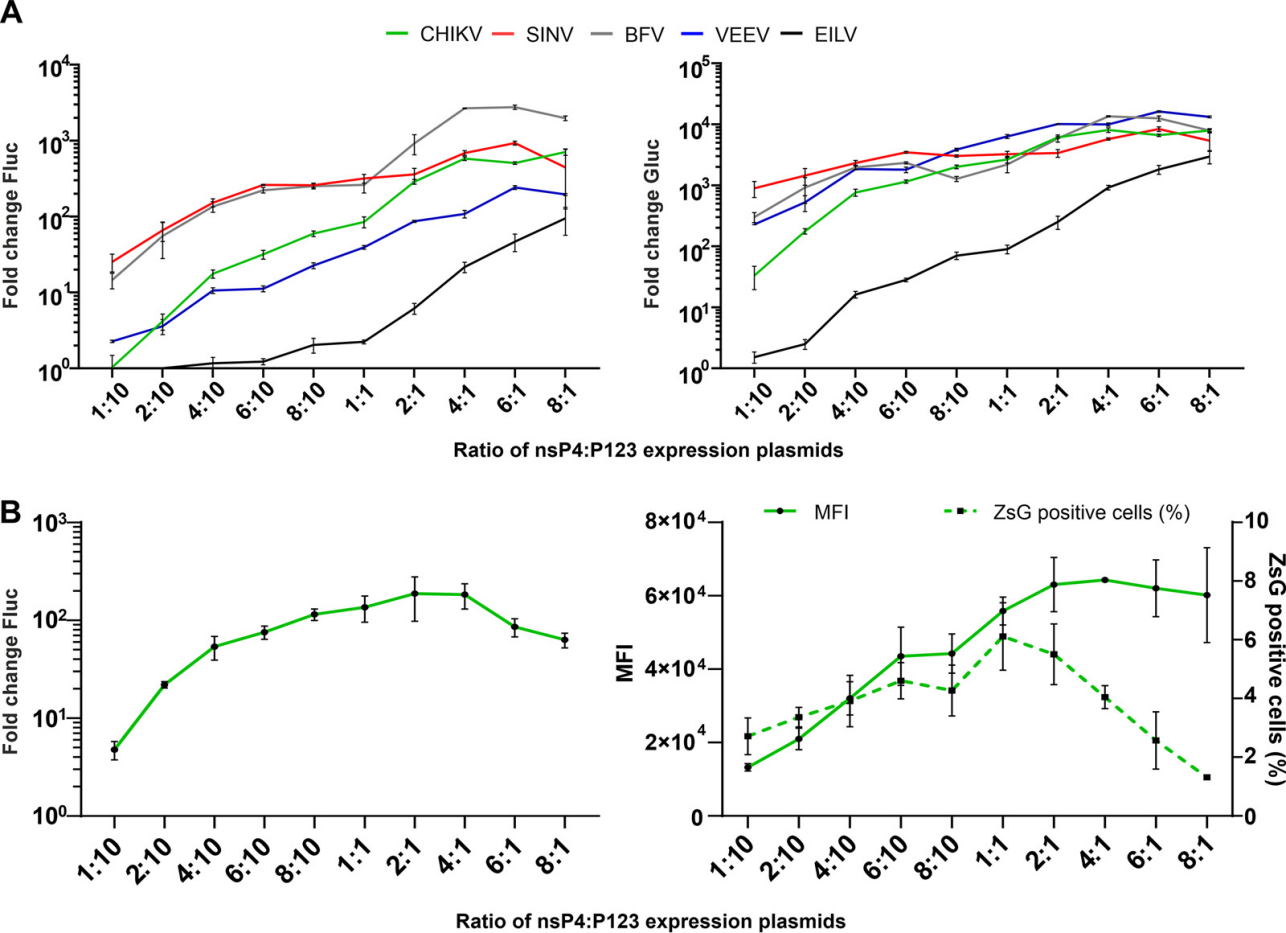
Activity of two-component replicase depends on the relative amount of nsP4. (A) HEK293T cells in 96-well plates were cotransfected with matching combinations of CMV-P123, CMV-ubi-nsP4, and HSPolI-FG plasmids of CHIKV, SINV, BFV, VEEV, and EILV. As negative controls, CMV-P1234^GAA^ was used instead of CMV-P123+CMV-ubi-nsP4. The amounts of CMV-P123 and HSPolI-FG were kept constant, while the CMV-ubi-nsP4 was provided at a 1:10, 2:10, 4:10, 6:10, 8:10, 1:1, 2:1, 4:1, 6:1, or 8:1 molar ratio in respect to CMV-P123. Cells were incubated and the data were collected and analyzed as described for Fig. 2B. Boosts of Fluc (replication, left panel) and Gluc (transcription, right panel) activities are shown as the means ± SD of three independent experiments. (B) HEK293T cells in 96-well plates (left) or in 24-well plates (right) were cotransfected with matching combinations of CMV-P123-CHIKV, CMV-ubi-nsP4-CHIKV, and HSPolI-FZsG-CHIKV. As the negative control, CMV-P1234^GAA^-CHIKV was used instead of CMV-P123+CMV-ubi-nsP4. The amounts of CMV-P123-CHIKV and HSPolI-FG-CHIKV were kept constant, while the CMV-ubi-nsP4-CHIKV plasmid was provided at a 1:10, 2:10, 4:10, 6:10, 8:10, 1:1, 2:1, 4:1, 6:1, or 8:1 molar ratio in respect to CMV-P123-CHIKV. (Left panel) Cells were incubated and the data were collected and analyzed as described for Fig. 2B, except that only Fluc activity (replication) was measured and shown as the means ± SD of three independent experiments. (Right panel) Cells were collected at 18 h p.t. and analyzed with an Attune NxT acoustic focusing cytometer. The percentage of ZsGreen-expression from living cells and mean fluorescence intensity (MFI) in arbitrary units for ZsGreen-positive cells are shown. The means ± SD of three independent experiments are shown.

The superior activities displayed by most of the two-component replicases were unexpected and, to the best of our knowledge, have not been previously observed. The obvious difference between single- and two-component replicases is the nsP4 to P123 ratio. For a single-component replicase, this ratio should be similar to that in alphavirus-infected cells. For two-component replicases, it may be different because for most alphaviruses, the synthesis of nsP4 is reduced due to the in-frame opal termination codon at the end of the region encoding nsP3. Furthermore, nsP4 is an unstable protein, and its stabilization requires interaction with P123 and/or its cleavage products (54, 55). We therefore hypothesized that the nsP4 to P123 ratio might be a determinant for the efficiency of viral RNA synthesis; this was verified using replicases of alphaviruses belonging to five different complexes, i.e., CHIKV, SINV, BFV, VEEV, and EILV. For the replicases of all of these viruses, a strong effect of the nsP4 to P123 ratio of expression plasmids on RNA synthesis was observed. At a 1:10 (nsP4:P123 expression plasmid) ratio, the boost of Fluc activity was close to or below the detection limit for CHIKV, VEEV, and EILV, but it was higher (approximately 10-fold) for BFV and SINV. At an 8:1 (nsP4:P123 expression plasmid) ratio, the boost of Fluc expression was increased by approximately 10- to 100-fold. The same trend was observed for the boost of Gluc expression except that the impact of higher nsP4 to P123 expression plasmid ratios on transcription activities of SINV, BFV, and VEEV replicases were somewhat less pronounced (Fig. 3A). Although the changes in ratios of expression plasmids do not necessarily result in identical changes in nsP4 to P123 protein ratios in transfected cells, these data clearly indicated that the amount of available nsP4 is the rate-limiting factor for functional replication formation/activity.

The observed increase of viral RNA synthesis in transfected cells can originate from an increased number of cells where RNA replication is initiated, from more efficient RNA synthesis in such cells or from a combined effect. To study this directly, we took advantage of a CHIKV template where the sequence encoding the Gluc reporter is substituted with a sequence encoding the fluorescent marker protein ZsGreen. In order to increase the percentage of ZsGreen-positive cells, which was essential for achieving a higher sensitivity for subsequent flow cytometry analysis, the transfections were performed using Lipofectamine LTX instead of FuGENE 6. It was observed that the boost of Fluc expression from a template encoded by HSPolI-FZsG-CHIKV was also strongly increased by the change in ratio of nsP4:P123 expression plasmids (Fig. 3B, left panel). Small differences between the profiles of Fluc expression in cells transfected using HSPolI-FG-CHIKV (Fig. 3A) or HSPolI-FZsG-CHIKV (Fig. 3B) may result from the use of different transfection reagents, from replacement of the sequence encoding Gluc with the sequence for ZsGreen, and/or from a possible impact of ZsGreen expression on the cell or on the activity of CHIKV RNA replicase. The flow cytometry analysis revealed that the increase in ratio of nsP4:P123 expression plasmids also resulted in a prominent increase of ZsGreen fluorescence in cells where the RNA replication was initiated. Compared with cells transfected using a 1:10 ratio of nsP4:P123 expression plasmids, the mean fluorescence intensity increased approximately 4.7-fold (*P* < 0.001) in cells transfected using a 2:1 ratio of these expression plasmids (Fig. 3B). At the same time, the ratio of nsP4:P123 expression plasmids also affected the percentage of cells in which RNA replication was initiated; i.e., the percentage of ZsGreen-positive cells peaked at a 1:1 ratio of nsP4:P123 expression plasmids and decreased at higher ratios (Fig. 3B). Most likely, this decline resulted from a toxic effect of increased amounts of nsP4 expression plasmid and/or from an adverse effect of high levels of ZsGreen on cells or on CHIKV RNA replicase activity. However, it cannot be excluded that very high nonphysiological levels of nsP4 can potentially also disturb the efficiency of replication complex formation.

Interestingly, it was observed that the impact of the nsP4 to P123 ratio on the activity of the EILV replicase was stronger than that of other replicases. At a 1:1 (nsP4:P123 expression plasmid) ratio, the EILV replicase had very low activities (Fig. 2B and C and 3A), while the increase in ratio to 8:1 (nsP4:P123 expression plasmid) resulted in highly significant increases of both template RNA replication (approximately 40-fold, *P* < 0.01) and transcription (approximately 30-fold, *P* < 0.01). These data suggest that the low activity of the EILV replicase in mammalian cells is mostly due to the properties of its nsP4 component. In addition, it is possible that expression of EILV nsP4 in mammalian cells was inefficient due to unfavorable codon usage of the ISV genome for mammalian cells and/or because of a reduced stability of EILV nsP4 in mammalian cells. Regardless of the exact nature of this defect, it could be partly compensated for by an increase in nsP4 expression.

### Functional replicases are formed by numerous heterologous combinations of P123 and nsP4 components.

The two-component replicase allows investigation of the functional compatibility of the P123 and nsP4 components belonging to different alphaviruses. Experimental testing of all possible combinations of CMV-P123, CMV-ubi-nsP4, and HSPolI-FG plasmids corresponding to nine different viruses was not feasible because of the vast numbers of possible combinations. However, we previously demonstrated that replicases of all alphaviruses examined here are capable of replicating and transcribing SINV template RNA (11), thereby providing a “universal” template for such studies. Thus, HEK293T cells were transfected using HSPolI-FG-SINV, as the source of template RNA, together with all combinations of CMV-P123 and CMV-ubi-nsP4 (used in equimolar ratios).

**FIG 4:**
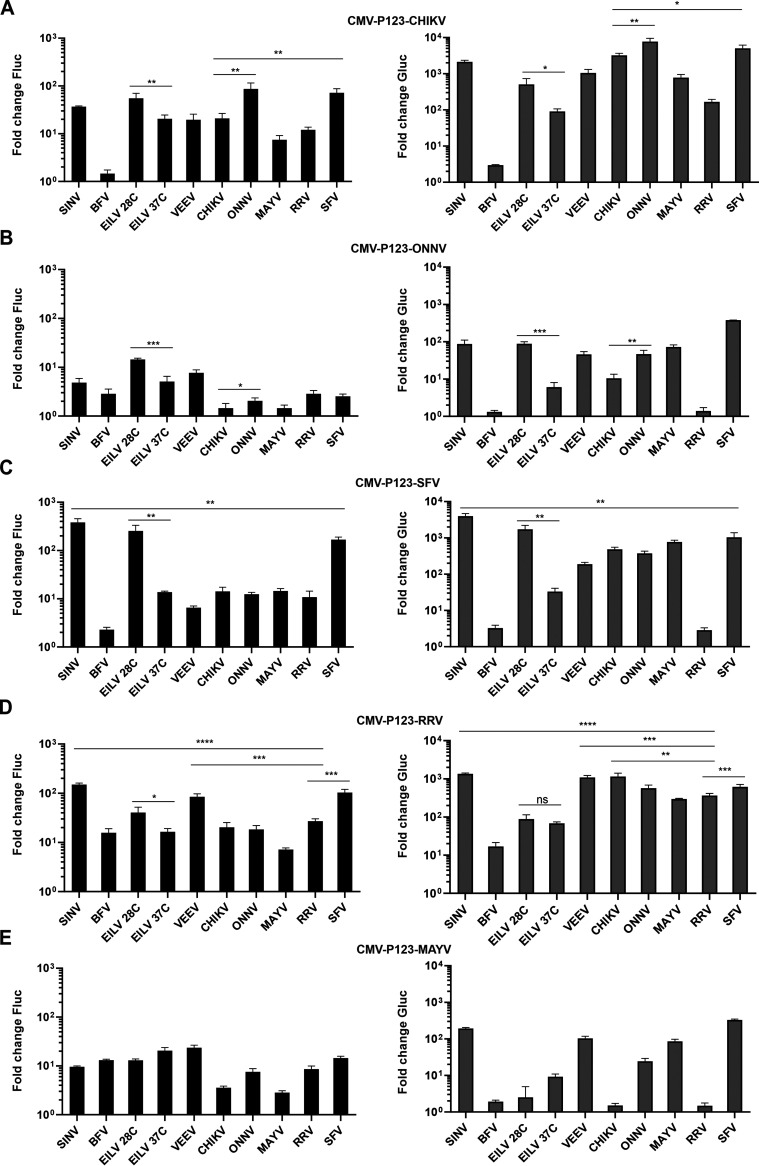
P123 components of viruses belonging to the SFV complex are compatible with heterologous nsP4 proteins. (A to E) HEK293T cells were cotransfected with HSPolI-FG-SINV and (A) CMV-P123-CHIKV, (B) CMV-P123-ONNV, (C) CMV-P123-SFV, (D) CMV-P123-RRV, or (E) CMV-P123-MAYV. In each transformation, one plasmid, expressing nsP4 from a virus shown on the *x* axes, was added in an equimolar amount to the indicated CMV-P123 plasmid. Transfected cells were incubated at 37°C and lysed for 18 h p.t.; in addition, cells transfected with CMV-ubi-nsP4-EILV were incubated at 28°C and lysed for 48 h p.t. The data represent the activity of Fluc and Gluc from CMV-ubi-nsP4-transfected cells normalized to the paired CMV-P1234^GAA^ control cells. Values obtained for P1234^GAA^ controls were taken as 1. The means ± SD from three independent experiments are shown. *, *P* < 0.05; **, *P* < 0.01; ***, *P* < 0.001; ****, *P* < 0.0001; n.s., not significant (Student’s unpaired *t* test).

It was observed that CHIKV P123 formed active replicases with nsP4 proteins of each alphavirus except BFV (Fig. 4A). Replicases containing the P123 of CHIKV and an nsP4 of ONNV or SFV were even more effective for RNA replication than the replicase containing the P123 and nsP4 of CHIKV (*P* < 0.01 in both cases); the same was the case for transcriptase activity (*P* < 0.01 and *P* < 0.05, respectively). Surprisingly, the replicase consisting of P123 of CHIKV and nsP4 of EILV was active at 37°C. Even though its activity was significantly higher at 28°C (*P* < 0.01 for replication; *P* < 0.01 for transcription) it is clear that the nsP4 of EILV is not only functional in mammalian cells, but also tolerates the higher temperatures typical of mammals. This conclusion was supported by the analysis of heterologous combinations involving nsP4 of EILV and P123 of ONNV, SFV, or RRV (Fig. 4B to D).

**FIG 5:**
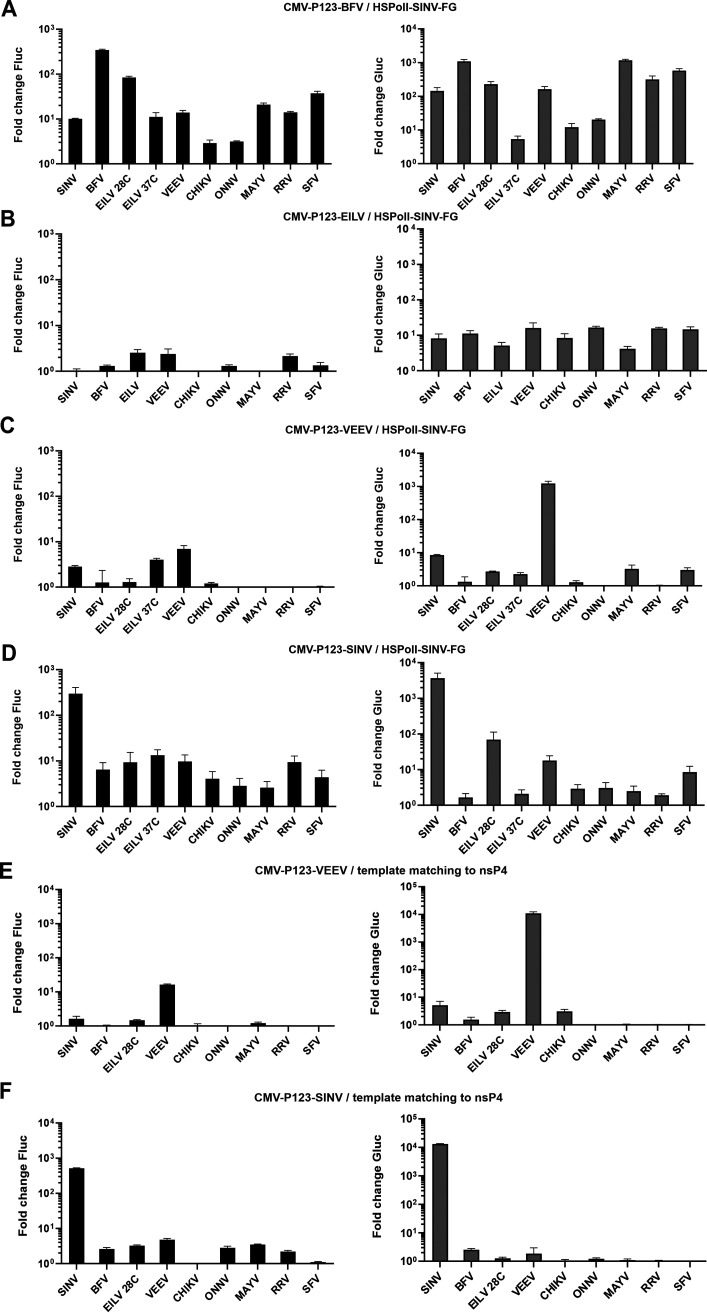
P123 components of outgroup alphaviruses are generally not compatible with heterologous nsP4 proteins. (A to D). HEK293T cells were cotransfected with HSPolI-FG-SINV and (A) CMV-P123-BFV, (B) CMV-P123-EILV, (C) CMV-P123-VEEV, or (D) CMV-P123-SINV. In each transformation, one plasmid, expressing nsP4 from a virus shown on the *x* axes, was added in an equimolar amount to the indicated CMV-P123 plasmid. Transfected cells were incubated at 37°C and lysed for 18 h p.t.; cells transfected with CMV-ubi-nsP4-EILV were also incubated at 28°C and lysed for 48 h p.t. All cells transfected with CMV-P123-EILV were incubated at 28°C and lysed for 48 h p.t. (E and F) HEK293T cells were cotransfected with (E) CMV-P123-VEEV or (F) CMV-P123-SINV and matching pairs of plasmids expressing nsP4 and template RNA from a virus shown on the *x* axes. Transfected cells were incubated at 37°C and lysed for 18 h p.t.; cells transfected with CMV-ubi-nsP4-EILV and HSPolI-FG-EILV were incubated at 28°C and lysed for 48 h p.t. In all experiments, data were collected, analyzed and presented as described for Fig. 4; activities lower than those observed for negative controls are also shown as 1. The means ± SD from three independent experiments are shown.

We have previously reported that in human cells, the *trans-*replicase of ONNV is considerably less active than those of other alphaviruses, including the *trans-*replicase of the closely related CHIKV (11). Here, an increased activity of replicase comprising P123 of CHIKV and nsP4 of the closely related ONNV was observed (Fig. 4A). In contrast, all *trans*-replicases containing P123 of ONNV had low activities (Fig. 4B). The activity of the replicase consisting of P123 of ONNV and nsP4 of CHIKV was significantly lower than that of the two-component ONNV replicase (*P* < 0.05 for replication; *P* < 0.01 for transcription). Furthermore, almost all combinations of nsP4 of ONNV with heterologous P123 had replication activities higher than those of two-component ONNV replicase (Fig. 4 and 5). Thus, the low activity of ONNV *trans-*replicase is clearly associated with properties of its P123 component. It was also observed that nsP4 of BFV or RRV combined with P123 of ONNV almost completely failed to transcribe the SINV template RNA (Fig. 4B). The same was the case for combinations of P123 of SFV and nsP4 of BFV and RRV. However, in contrast to P123 of ONNV, P123 of SFV formed highly active replicases with nsP4 components of other alphaviruses (Fig. 4C). The activity of replicase consisting of P123 of SFV and nsP4 of SINV even exceeded that of the two-component SFV replicase (*P* < 0.01 both for replication and transcription). The P123 of RRV was also able to form several heterologous combinations that were more active than the two-component replicase of RRV (Fig. 4D). The increased replication activity was obtained by combining RRV P123 with nsP4 of SINV, VEEV, or SFV (*P* < 0.001 in all cases), while increased transcriptase activities were observed for combinations containing nsP4 of SINV, VEEV, SFV (*P* < 0.001 in all cases), or CHIKV (*P* < 0.01). Consistent with our observation that the two-component replicase of MAYV has a relatively low activity (Fig. 2B to D), the combinations of P123 of MAYV with heterologous nsP4 proteins resulted in replicases having limited activities (Fig. 4E). At the same time, nsP4 of MAYV formed significantly more active replicases with P123 of CHIKV and SFV than with matching P123 (for transcription *P* < 0.01 and *P* < 0.0001, respectively). Thus, similar to ONNV, the low activity of the two-component replicase of MAYV can be attributed to its P123 component.

From outgroup alphaviruses, only P123 of BFV was capable of forming relatively active replicase complexes with nsP4 proteins belonging to other alphaviruses. The lowest activities were observed for combinations of P123 of BFV and nsP4 of the closely related CHIKV or ONNV (Fig. 5A). It is noteworthy that the nsP4 of BFV was the only nsP4 protein analyzed in these studies that failed to form a functional replicase with any P123 tested other than that from BFV itself. None of the heterologous combinations had a replication activity higher than 5% and transcription activity higher than 2% of that of matching combination (Fig. 4 and 5). In contrast to the P123 of BFV, the P123 of EILV failed to form an active replicase with any of the nsP4s, as evidenced by the very modest boost of Fluc (which did not exceed 3-fold) and Gluc (up to 20-fold) activities (Fig. 5B). In this regard, the P123 of EILV was similar to the P123 of ONNV and MAYV (compare Fig. 5B with Fig. 4B and E). However, for EILV P123, the failure to form a functional replicase does not necessarily indicate a functional incompatibility, as a large excess of nsP4 is necessary to compensate for an *in vitro* defect even for the homologous two-component system of EILV (Fig. 3A). Interestingly, similar to ONNV and MAYV, the nsP4 of EILV formed more active replicases with P123 components from many heterologous alphaviruses (Fig. 4 and 5).

P123 of VEEV and SINV displayed nearly identical properties. Both of them formed highly active replicases with the matching nsP4 component but essentially failed to do so with any of the heterologous nsP4 proteins (Fig. 5C and D). However, VEEV *trans-*replicase was previously shown to have reduced activity on a SINV RNA template (11). To exclude the possibility that the observed incompatibility of P123 of VEEV and nsP4 proteins of heterologous alphaviruses was due to a suboptimal template, HEK293T cells were also transfected with CMV-P123-VEEV and matching combinations of nsP4 and template RNA expression plasmids. Again, only the combination of VEEV P123 and nsP4 resulted in active replication and transcription of the provided template RNAs (Fig. 5E). Almost identical results were obtained when the same experiment was performed using CMV-P123-SINV. In contrast to the highly active two-component replicase of SINV, no heterologous combination containing SINV P123 boosted Fluc expression more than 4-fold or Gluc expression more than 3-fold (Fig. 5F). Thus, the P123 components of SINV and VEEV were not functionally compatible with nsP4 of other alphaviruses in the *trans*-replication assay. This contrasts with the properties of their nsP4 components that form functional replicases with P123 components of several heterologous alphaviruses (Fig. 4 and 5).

### SFV tolerates the replacement of nsP4 with its counterpart from other alphaviruses, and for SINV, similar exchange results in viruses with temperature-sensitive phenotype.

**FIG 6:**
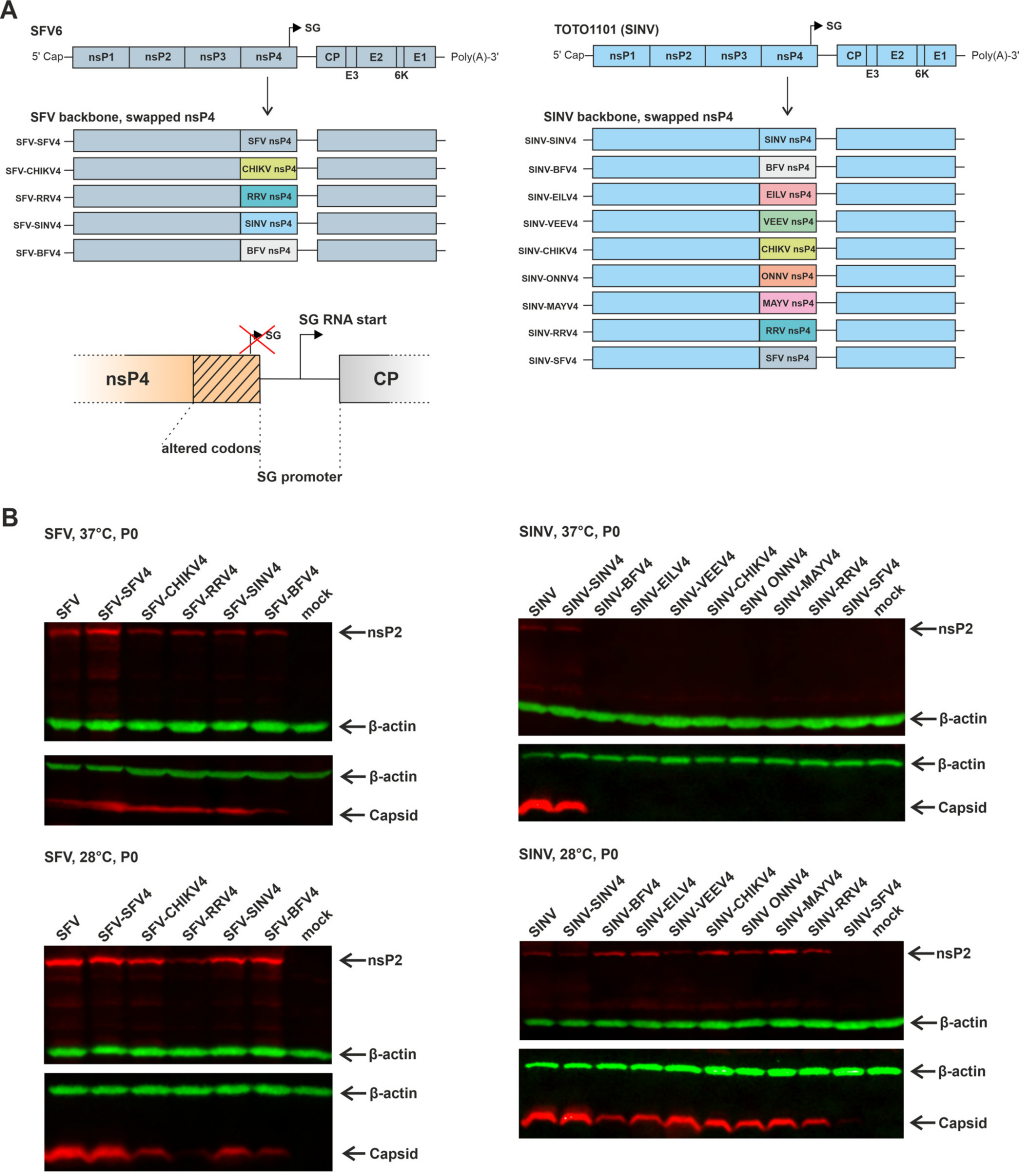
Properties of chimeric SFV and SINV harboring heterologous nsP4. (A) Schematic presentation of chimeric SFV (left) and SINV (right) genomes. Swapped nsP4 regions are color-codes. The modified junction region between ns- and structural ORFs, including the codon-altered region of nsP4 (Table 1), is shown below the drawing of chimeric SFV genomes. The arrow indicates the transcription start site of the SG promoter of the virus. The red X indicates inactivation of the SG promoter by synonymous mutations introduced into the sequence encoding the C-terminal region of nsP4. (B) The lysates of the BHK-21 cells transfected with icDNA plasmids of SFV and its chimeras (left panels) or with *in vitro* transcripts of icDNA of SINV and its chimeras (right panels) and lysate from mock-transfected control cells were subjected to SDS-PAGE and immunoblot analysis with antibodies against the corresponding nsP2 and capsid proteins. β-actin is shown as a loading control. The top panels show lysates of transfected cells that were incubated at 37°C, while the bottom panels show lysates of transfected cells that were incubated at 28°C. The experiment was repeated twice with similar results; the data from one experiment are shown.

The *trans-*replication assay revealed that the P123 of SFV forms an efficient RNA replicase with the nsP4 of SINV or CHIKV. At the same time, replication and transcription activities of replicases formed with the nsP4 of RRV or BFV were much lower (Fig. 4C). To verify these findings in the context of the virus genome, chimeric SFV genomes were constructed where the nsP4 of SFV was replaced with that of SINV, CHIKV, RRV, or BFV (Fig. 6A). As the SG promoter of alphaviruses mostly resides inside the region encoding the C-terminal part of nsP4, the cloning was performed in such a way that the native SG promoter of SFV (from position −98 with respect to the SG RNA start site to the end of the intergenic region) was maintained, while the heterologous SG promoters residing inside the nsP4 region of SINV, CHIKV, RRV, or BFV were erased using synonymous substitutions (Fig. 6A; Table 1). To account for the effect caused by this cloning strategy, a control virus designated SFV-SFV4, where the native nsP4 region was replaced with nsP4 of SFV modified in the same manner, was also constructed. The infectious center assay (ICA) performed in BHK-21 cells confirmed high infectivity of infectious cDNA (icDNA) plasmids of wild-type (WT) SFV6 (56). The infectivity of icDNA of SFV-SFV4 was similar (Table 2), indicating that rearrangements made in the SG promoter region had little or no impact on virus rescue and infectivity. ICA did not reveal any plaques for icDNAs of SFV-CHIKV4, SFV-RRV4, SFV-SINV4, or SFV-BFV4 (Table 2). However, when the transfected cells were plated and incubated at 37°C, the development of cytopathic effect (CPE) was observed. CPE appeared earlier and was most prominent for SFV-CHIKV4 and SFV-SINV4, whereas it developed slower and was less prominent for SFV-RRV4 and SFV-BFV4 (Table 2). Thus, the time and extent of CPE development reflected the activities of the corresponding heterologous *trans-*replicases (Fig. 4C). Interestingly, when the transfected cells were incubated at 28°C, only a very mild CPE was observed for all four SFV chimeras (Table 2). The rescue of viruses was, however, successful at both temperatures, and both nsP2 and capsid protein of SFV were clearly detected in lysates of transfected cells (Fig. 6B). Therefore, it was concluded that the lack of prominent CPE at 28°C was not due to the lack of virus rescue. The infection of cells, performed using harvested virus stocks, confirmed that these chimeric viruses were cytotoxic at 37°C but had no or very limited cytotoxicity at 28°C (Table 2). The reduced cytotoxicity may explain the failure of chimeric SFV constructs to form plaques in the ICA. It was also observed that SFV-RRV4 expressed only very low levels of capsid protein at 28°C. This correlates with the finding made using a *trans*-replicase consisting of P123 from SFV and nsP4 from RRV and may indicate that the aberrant utilization of the SG promoter found for replicases consisting of nsP4 from RRV and P123 from SFV, ONNV, or MAYV (Fig. 4B, C, and E) may not be specific for the SG promoter of SINV and extends to several heterologous SG promoters.

**TABLE 1:** Native and codon-modified sequences encoding the C-terminal region of the nsP4 protein of nine alphaviruses

Virus	Native or modified	Sequence
SFV	Native	GAGGTAGAGGGCTGCAAAAGTATCCTCATAGCCATGGCCACCTTGGCGAGGGACATTAAGGCGTTTAAGAAATTGAGAGGACCTGTTATACACCTCTACGGCGGTCCTAGATTGGTGCGT
	Modified	GAGGTgGAaGGCTGCAAgAGcATCCTgATcGCCATGGCCACacTGGCcAGaGACATcAAGGCcTTcAAGAAAcTGAGAGGcCCcGTgATcCACCTgTACGGCGGaCCTAGAcTcGTGCGg
CHIKV	Native	TCTAGGTACGAAGTGCAGGGTATATCAGTTGTGGTAATGTCCATGGCCACCTTTGCAAGCTCCAGATCCAACTTCGAGAAGCTCAGAGGACCCGTCATAACTTTGTACGGCGGTCCTAAA
	Modified	TCTAGaTACGAgGTGCAGGGcATcagcGTgGTGGTcATGagCATGGCCACCTTcGCcAGCagCAGAagCAACTTCGAGAAGCTgAGAGGcCCCGTgATcACccTGTAtGGCGGaCCTAAA
ONNV	Native	TCAAGGTATGAAGTACAAGGAATAACAGCCGTAATAACATCAATGGCTACCTTTGCGAGTAGCAAAGAAAACTTTAAAAAACTAAGAGGGCCCGTCGTAACCTTGTACGGCGGACCTAAA
	Modified	TCAAGaTAcGAgGTgCAgGGcATcACcGCCGTgATcACcagcATGGCcACaTTcGCcAGcAGCAAAGAgAACTTcAAgAAACTgAGAGGcCCCGTCGTgACCcTGTAtGGCGGACCTAAA
RRV	Native	TCTCGGTATGAGGTGAACGGGACCGGCAACATAGTGCGAGCAATGGCCACACTGGCCAAGAGCCTGAAGAATTTTAAAAAGCTGCGTGGACCCATCGTACACCTCTACGGCGGTCCTAAA
	Modified	TCTCGcTAcGAaGTGAACGGcACCGGCAACATcGTcaGAGCcATGGCCACACTGGCCAAGAGCCTGAAGAAcTTcAAgAAGCTGaGaGGcCCCATCGTgCACCTgTAtGGCGGaCCTAAA
MAYV	Native	ACCAGATACGAGGTGGAAGGGGGTTACAACCTATTGTTGGCTATGTCCACCTTTGCACACAGTATGAAGAATTTTTCTGCATTGAGGGGACCCGTCATACACTTGTACGGCGGTCCTAAA
	Modified	ACCcGcTAtGAGGTGGAAGGcGGcTACAAtCTgcTGcTGGCcATGagCACaTTcGCcCACAGcATGAAGAAcTTcagcGCccTGAGaGGcCCCGTgATcCAtcTttAtGGCGGcCCTAAg
SINV	Native	ACCCGGTATGAGGTAGACAATATTACACCTGTCCTACTGGCATTGAGAACTTTTGCCCAGAGCAAAAGAGCATTCCAAGCCATCAGAGGGGAAATAAAGCATCTCTACGGTGGTCCTAAA
	Modified	ACCaGaTAcGAGGTgGACAAcATcACcCCTGTgCTgCTGGCccTGAGAACaTTcGCCCAGAGCAAgAGAGCcTTCCAgGCCATCAGAGGcGAgATcAAGCAcCTgTAtGGcGGcCCTAAg
BFV	Native	GACCGTTATGCCGTCCACTCATCAGAACTAGTTTTATTGGCACTGACTACTCTGTCTAAGAACTTGAAGTCCTTCAGAAACATAAGAGGGAAACCAATACATCTCTACGGTGGTCCTAAA
	Modified	GACaGaTAcGCCGTgCACTCtTCtGAgCTgGTgcTgcTGGCtCTGACcACaCTGagcAAGAACcTGAAGTCCTTCcGgAACATccGgGGcAAgCCcATcCATCTgTAtGGcGGaCCTAAg
VEEV	Native	GCAGTAGAATCAAGGTATGAAACCGTAGGAACTTCCATCATAGTTATGGCCATGACTACTCTAGCTAGCAGTGTTAAATCATTCAGCTACCTGAGAGGGGCCCCTATAACTCTCTACGGC
	Modified	GCAGTcGAgagcAGaTAcGAgACaGTgGGcACcagCATCATcGTgATGGCCATGACaACaCTgGCcAGCAGcGTgAAgTCcTTCAGCTACCTtAGAGGcGCCCCTATcACaCTgTACGGC
EILV	Native	GCCGTATCATCAAGATACGAAGTCGACAACATACTGCCCGTTCTCTTAGCCCTTAGAACCTTTGCTTTATCTACGCGCAACTTCTCTGCCTTACGGGGAACACTTAAGACCCTCTACAAC
	Modified	GCCGTgTCtagcAGATACGAgGTgGACAACATcCTGCCtGTgCTgcTgGCCCTgAGAACaTTTGCccTgagcACcaGaAACTTCagcGCCcTgaGaGGcACcCTgAAaACCCTgTACAAC

**TABLE 2:** Properties of chimeric viruses^*a*^

icDNA	Transfections								
	37°C			28°C		Infections with p0 stock			
	ICA	Virus collected		Virus collected		37°C		28°C	
	PFU/μg	h p.t.	CPE	h p.t.	CPE	h p.i.	CPE	h p.i.	CPE
SFV6	250,000	24	Cell death	48	Cell death	24	Cell death	48	Cell death
SFV-SFV4	200,000	24	Cell death	48	Cell death	24	Cell death	48	Cell death
SFV-CHIKV4	<2	72	Strong	96	Very mild^*b*^	24	Cell death	72	Very mild^*b*^
SFV-RRV4	<2	72	Mild	96	Very mild^*b*^	24	Cell death	72	Very mild^*b*^
SFV-SINV4	<2	72	Strong	96	Very mild^*b*^	24	Cell death	72	Very mild^*b*^
SFV-BFV4	<2	72	Medium	96	Very mild^*b*^	24	Cell death	72	Very mild^*b*^
SINV (Toto1101)	80,000	24	Cell death	48	Cell death	24	Cell death	48	Cell death
SINV-SINV4	80,000	24	Cell death	48	Cell death	24	Cell death	48	Cell death
SINV-BFV4	<2	72	Not observed	48	Strong	72	Not observed	48	Cell death
SINV-EILV4	<2	72	Not observed	72	Strong	72	Not observed	48	Cell death
SINV-VEEV4	<2	72	Not observed	96	Mild	72	Not observed^*c*^	48	Cell death
SINV-CHIKV4	<2	72	Not observed	72	Strong	72	Not observed	48	Cell death
SINV-ONNV4	<2	72	Not observed	48	Strong	72	Not observed	48	Cell death
SINV-MAYV4	<2	72	Not observed	48	Strong	72	Not observed	48	Cell death
SINV-RRV4	<2	72	Not observed	96	Medium	72	Not observed	48	Cell death
SINV-SFV4	<2	72	Not observed	96	Not observed	72	Not observed	48	Cell death

a Data from one experiment out of two are shown.

b Signs of CPE were observed 24 h before virus stock was collected, but CPE did not progress and no cell death was observed.

c In one experiment, a slight change of color of growth medium was observed.

Unlike the P123 of SFV, the P123 of SINV is unable to form an efficient replicase with any of the heterologous nsP4 proteins (Fig. 5D and F). To analyze if this property is also maintained in the context of virus genome, nine chimeras, including the SINV-SINV4 control (Fig. 6A), were constructed using the above-described approach. Only the transcripts corresponding to WT SINV and SINV-SINV4 were able to form plaques in ICA (Table 2). In contrast to the SFV chimeras, when cells transfected with transcripts corresponding to SINV-BFV4, SINV-EILV4, SINV-VEEV4, SINV-CHIKV4, SINV-MAYV4, SINV-RRV4, or SINV-SFV4 were incubated at 37°C, CPE was not observed. However, the lack of CPE does not necessarily indicate a lack of replication. To address this, a Western blot analysis was done for nsP2 (as a marker of genomic RNA synthesis) and for capsid protein (as a marker of SG RNA synthesis). This analysis confirmed the absence of virus rescue, as we were unable to detect SINV nsP2 or capsid protein in the lysate of these cells (Fig. 6B). Thus, the combinations of SINV P123 with heterologous nsP4 proteins, which have no or very low RNA synthesis activity in the *trans*-replication assay (Fig. 5B and F), also resulted in noninfectious SINV chimeras. However, when the cells transfected with these transcripts were incubated at 28°C, the development of CPE was observed. In general, CPE was strong except in cells transfected with SINV-VEEV4 and SINV-RRV4 transcripts. No CPE was observed in cells transfected with SINV-SFV4 transcripts (Table 2). Immunoblot analysis confirmed the rescue of chimeric viruses. The levels of SINV nsP2 and capsid protein were lowest (but clearly detectable) in cells transfected with SINV-SFV4 (Fig. 6B). The collected chimeric viruses were unable to infect BHK-21 at 37°C; however, they were infectious and caused CPE when infected cells were incubated at 28°C (Table 2). These data demonstrate that in the context of viral genome and in BHK-21 cells, the combination of SINV P123 with heterologous nsP4 results in viruses with a strong temperature-sensitive phenotype.

### Two- and three-component replicases containing protease-negative P12^CA^3 or P2^CA^3 are functional.

Nonprocessed P123 is a key component of the negative-strand replicase of alphaviruses (20). However, it has been shown that a SINV mutant, unable to process P123 and containing a Glu451 to Ala adaptive change in nsP4, is viable and capable of efficient synthesis of new genomic and SG RNAs (28). The *trans*-replicase of CHIKV, unable to process P123, has also been shown to synthesize positive-strand RNAs (57), and the same has been observed for two-component replicases of SFV and SINV containing Cys to Ala mutations in the active site of nsP2 protease (49).

**FIG 7:**
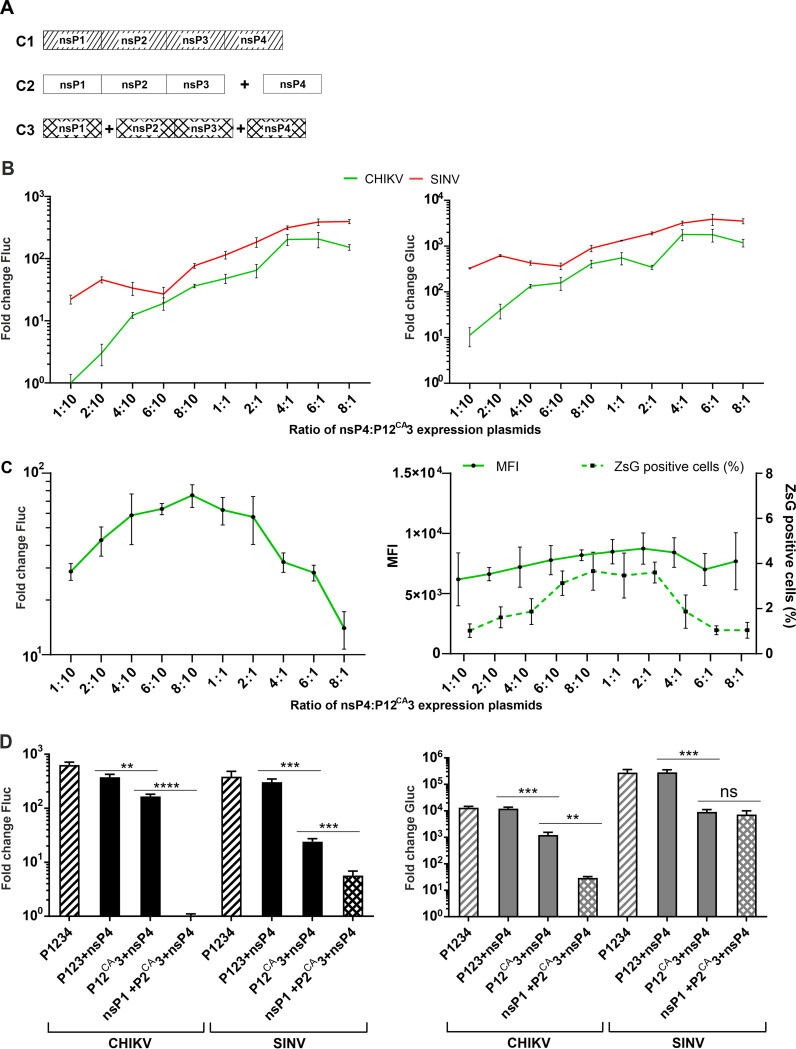
Two- and three-component replicases of SINV and CHIKV containing inactive protease are capable of RNA synthesis. (A) Schematic presentation of the replicases used. C1 shows the P1234 expressed from a single-component CMV-P1234 plasmid, C2 shows the P123 and nsP4 expressed by the two-component replicase, and C3 shows nsP1, P2^CA^3 and nsP4 expressed by the three-component replicase. (B). HEK293T cells in 96-well plates were cotransfected with CMV-P12^CA^3-CHIKV, CMV-ubi-nsP4-CHIKV, and HSPolI-FG-CHIKV or CMV-P12^CA^3-SINV, CMV-ubi-nsP4-SINV, and HSPolI-FG-SINV plasmids. As the negative controls, CMV-P1234^GAA^, which lack polymerase activity, were used. The amounts of CMV-P12^CA^3 and HSPolI-FG plasmids were kept constant, while the CMV-ubi-nsP4 plasmid was provided in a 1:10, 2:10, 4:10, 6:10 8:10, 1:1, 2:1, 4:1, 6:1, or 8:1 molar ratio in respect to CMV-P12^CA^3. Cells were incubated and the data were collected and analyzed as described for Fig. 2B. Boosts of Fluc (replication, left panel) and Gluc (transcription, right panel) activities are shown as the means ± SD of three independent experiments. (C). HEK293T cells in 96-well plates (left) or in 24-well plates (right) were cotransfected with matching combinations of CMV-P12^CA^3-CHIKV, CMV-ubi-nsP4-CHIKV, and HSPolI-FZsG-CHIKV. As the negative control, CMV-P1234^GAA^-CHIKV was used instead of CMV-P12^CA^3+CMV-ubi-nsP4. This experiment was performed and the data were collected, analyzed, and presented as described for Fig. 3B. (D) U2OS cells in 12-well plates were cotransfected with single-, two-, and three-component replicases as indicated on the *x* axes. For two- and three-component replicases, all plasmids encoding ns-proteins were combined in equimolar amounts. As negative controls, CMV-P1234^GAA^ were used instead of CMV-P1234. Cells were incubated at 37°C and lysed for 18 h p.t. Fluc (replication, left panel) and Gluc (transcription, right panel) activities produced by active replicases were normalized to the P1234^GAA^ controls. The values obtained for the P1234^GAA^ controls were taken as 1. The means ± SD of three independent experiments are shown; **, *P* < 0.01; ***, *P* < 0.001; ****, *P* < 0.0001; n.s., not significant (Student’s unpaired *t* test).

Here, the Cys478 to Ala mutation was introduced in nsP2 of CHIKV and the Cys481 to Ala mutation was introduced in nsP2 of SINV. The ability of the obtained P12^CA^3 components of CHIKV and SINV to form a functional RNA replicase with the matching nsP4 component was analyzed across nsP4 to P12^CA^3 expression plasmid ratios from 1:10 to 8:1. Similarly to the corresponding WT two-component replicases, the replication and transcription activities of two-component P12^CA^3-containing replicases on template RNA encoding Fluc and Gluc reporters increased with increasing amounts of nsP4. Regardless of the protease activity of nsP2, the observed increase was more prominent for the two-component replicase of CHIKV (compare Fig. 3A and Fig. 7B). The results obtained using CMV-P12^CA^3-CHIKV, CMV-ubi-nsP4-CHIKV, and HSPolI-FZsG-CHIKV as source of RNA template were somewhat different. An increase in the nsP4 to P12^CA^3 expression plasmid ratio from 1:10 to 8:10 resulted in a modest increase of Fluc activity, while ratios higher than 2:1 resulted in a clear decline of Fluc activity (Fig. 7C). Most likely, this was caused by two overlapping effects. First, the percentage of cells where RNA replication was initiated was approximately 1.5-fold lower than in the case of P123+nsP4 replicase. Furthermore, the ZsGreen-positive cell percentage varied with changes in the ratio of nsP4:P123 protein expression plasmids in a similar way as the Fluc activity (Fig. 7C). Second, the increase in the ratio of nsP4 to P12^CA^3 expression plasmid had no significant effect on the intensity of the ZsGreen fluorescence (Fig. 7C). Therefore, the impact of the reduction in percentage of cells where replication was initiated was not compensated for by the increased replicase activity in these cells. Thus, although the formation of a functional replicase containing P12^CA^3 component generally followed the same rules as the two-component replicase containing WT P123, it was more sensitive to changes in the experimental conditions, such as a change in transfection reagent and the use of different reporters encoded by the template RNA. It is therefore plausible that some properties of the replicase complexes locked at the stage of early replicase (unprocessed P123+nsP4) are different from these of complexes that can be converted into mature replicase (nsP1+nsP2+nsP3+nsP4).

It has also been described that the replicase activity and the induction of spherule formation is maintained upon the additional split of P12^CA^3 into nsP1 and P2^CA^3 components (49). As the swapping of P123 and nsP4 components was found to be an efficient approach to study the compatibility of P123 and nsP4 components that belong to different alphaviruses, we hypothesized that the same approach may be extended to nsP1 and P23 components as well. To study this possibility, three-component replicases (Fig. 1A and 7A) were constructed for CHIKV, SINV, and RRV, and their activities were compared to those of the single-component replicase, the WT two-component replicase, and the two-component replicase with a mutation in the active site of nsP2. In these experiments, all components of the two- and three-component replicases were used in equimolar amounts compared to CMV-P1234 of the single-component replicase. In order to increase sensitivity, the experiment was performed using U2OS cells that have been found to be optimal for CHIKV and SINV *trans*-replicases (11, 26). As no detectable activity was observed for the three-component replicase of RRV (data not shown), this was excluded from further analysis. For CHIKV and SINV, it was found that the inactivation of the nsP2 protease activity resulted in a significant decrease of RNA replication and transcription activities of the two-component replicases, the effect being somewhat more prominent for SINV replicase (Fig. 7D). Splitting of P12^CA^3 into nsP1 and P2^CA^3 resulted in almost complete inhibition of RNA replication and significant reduction of transcription activities of CHIKV replicase. In contrast, the effect of splitting of P12^CA^3 on the RNA replication activity SINV replicase was more modest; no significant effect on the Gluc expression (transcription) was observed (Fig. 7D). Thus, the activities of the three-component replicases of SINV and CHIKV were clearly different. Interestingly, unlike the experiments using two-component replicases (Fig. 3 and 7B), our attempts to improve the performance of the three-component replicases of CHIKV and SINV by altering the ratios of nsP1, P2^CA^3, and nsP4 component-encoding plasmids were unsuccessful. Coupled with a lack of activity of the three-component replicase of RRV, these findings excluded the possibility of using three-component replicases for the analysis of functional compatibility of nsP1 and P2^CA^3 components belonging to different alphaviruses.

### For viruses belonging to the SFV complex, the activity of SG RNA synthesis is correlated with the nsP4 component.

The determinants necessary for the efficient replication of template RNA by alphavirus replicases have been mapped to the 5′ region of the genome. For SINV, these include the extreme 5′ end of the RNA genome and sequences corresponding to the first stem-loop structure in the 5′ untranslated region (11). However, to the best of our knowledge it has not been directly demonstrated which component of the RNA replicase—P123 or nsP4—is responsible for template preference. Here, we found that the P123 components of outgroup viruses are poorly (or not at all) compatible with the nsP4 components of heterologous alphaviruses (Fig. 5). In contrast, for several pairs of viruses belonging to the SFV complex, reciprocal combinations of P123 and nsP4 were shown to have high-level RNA replication and transcription activities (Fig. 4), allowing analysis of which replicase component is responsible for the RNA template preference.

**FIG 8:**
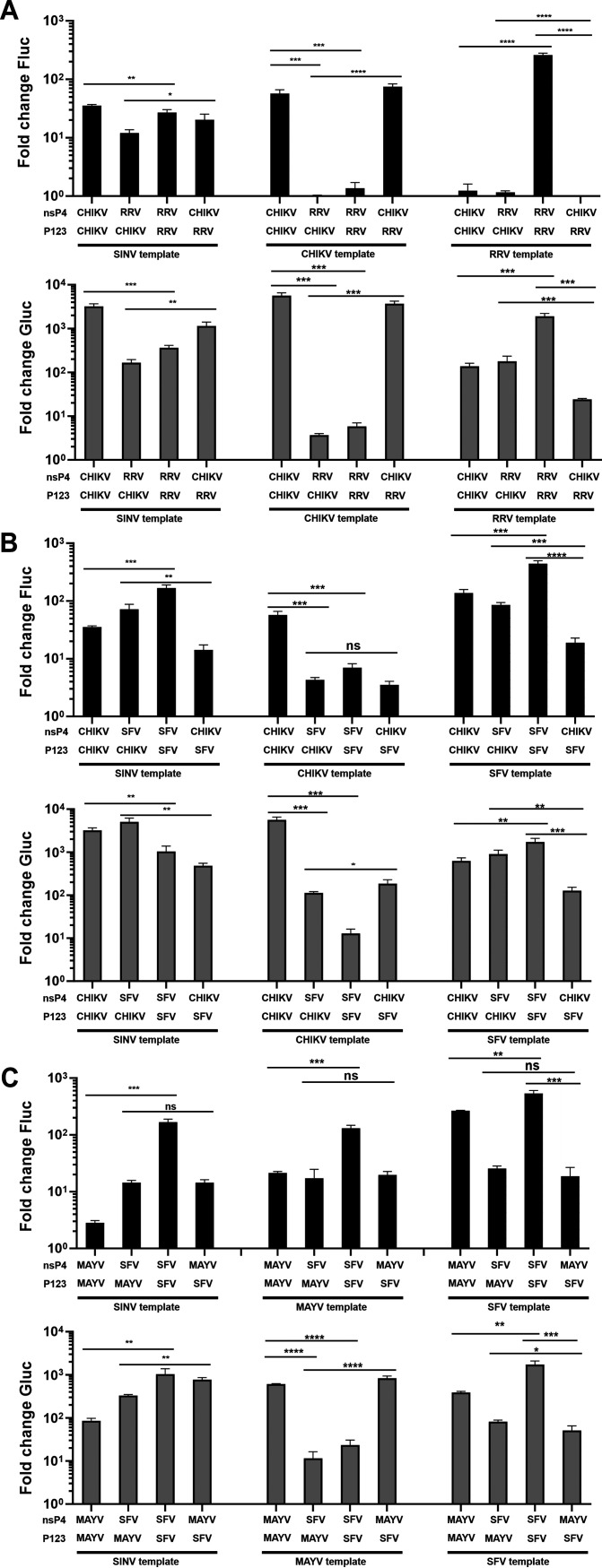
Template specificity of replicases of alphaviruses belonging to the SFV complex is associated with the nsP4 component. HEK293T cells in 96-well plates were cotransfected with (A) matching and heterologous combinations of CMV-P123-CHIKV or CMV-P123-RRV and CMV-ubi-nsP4-CHIKV or CMV-ubi-nsP4-RRV. Plasmids encoding template RNA were HSPolI-FG-SINV (left), HSPolI-FG-CHIKV (middle), or HSPolI-FG-RRV (right); (B) matching and heterologous combinations of CMV-P123-CHIKV or CMV-P123-SFV and CMV-ubi-nsP4-CHIKV or CMV-ubi-nsP4-SFV. Plasmids encoding template RNA were HSPolI-FG-SINV (left), HSPolI-FG-CHIKV (middle), or HSPolI-FG-SFV (right); (C) matching and heterologous combinations of CMV-P123-MAYV or CMV-P123-SFV and CMV-ubi-nsP4-MAYV or CMV-ubi-nsP4-SFV. Plasmids encoding template RNA were HSPolI-FG-SINV (left), HSPolI-FG-MAYV (middle), or HSPolI-FG-SFV (right). (A to C). CMV-P123 and CMV-ubi-nsP4 were always used in a 1:1 molar ratio. As negative controls CMV-P1234^GAA^ were used. Cells were incubated at 37°C and lysed for 18 h.p.t. In all experiments, data were collected, analyzed, and presented as described for Fig. 4; activities lower than those observed for negative controls are shown as 1. *, *P* < 0.05; **, *P* < 0.01; ***, *P* < 0.001; ****, *P* < 0.0001; n.s., not significant (Student’s unpaired *t* test).

On the SINV template, both heterologous combinations of P123 and nsP4 components of CHIKV and RRV were slightly less active than matching combinations. Consistent with previous observations using a single-component replicase (11), the two-component replicase of RRV had significantly lower activity on the CHIKV template, while the two-component replicase of CHIKV had significantly lower activity on the RRV template. The replicase formed by the P123 of RRV and the nsP4 of CHIKV was highly active on CHIKV template but replicated and transcribed the RRV template poorly (Fig. 8A). The replicase consisting of the P123 of CHIKV and the nsP4 of RRV replicated both CHIKV and RRV templates very poorly, but at the same time, it transcribed the RRV template more efficiently than the CHIKV template. Taken together, these data show that for RRV and CHIKV both replication and transcription activities were always significantly higher when the nsP4 component of replicase and RNA template belonged to the same virus (Fig. 8A). Thus, for RRV and CHIKV replicases, the preferences of genomic and SG promoters are associated with nsP4 proteins.

A similar trend was also revealed for replicase components of CHIKV and SFV. On SINV template, SFV replicase was significantly more active for its replication, while CHIKV replicase was significantly more active for its transcription; from heterologous combinations, significantly higher replication and transcription activities were observed for the replicase consisting of CHIKV P123 and SFV nsP4. The two-component replicase of SFV favored the matching template for both replication and transcription, while for two-component replicase of CHIKV, this was observed only for transcription (Fig. 8B). For replication of the CHIKV template, the replicase formed by the P123 of CHIKV and the nsP4 of SFV had activity similar to that of the reciprocal combination; it also significantly outperformed the latter on the SFV template (Fig. 8B). However, as the replicase formed by the P123 of CHIKV and the nsP4 of SFV was also more active on SINV template (Fig. 8B), it remains uncertain whether the observed superior activity on the SFV template was due to intrinsically higher replication activity of the complex and/or due to match between nsP4 and template RNA. For transcription, however, both templates were significantly more efficiently used when the nsP4 components of replicase and template RNA were from the same virus (Fig. 8B) again indicating the importance of a match between the nsP4 and SG promoter in the template RNA.

Similar trends were also observed for replicase components of MAYV and SFV. On the SINV template, the two-component replicase of SFV was significantly more active than the two-component replicase of MAYV (Fig. 8C). For replication, both two-component replicases used the SFV template more efficiently than the MAYV template, and the replicase of SFV outperformed that of MAYV on both templates (Fig. 8C). Replicases comprising heterologous P123 and nsP4 components had similar replication activities on all three templates (Fig. 8C). In contrast, template RNAs of SFV and MAYV were transcribed significantly more efficiently if there was a match between an RNA template and nsP4 of replicase consisting from heterologous components (Fig. 8C). Thus, the nsP4 component also has a leading role in recognition and use of SG promoters of MAYV and SFV. Combined, our data demonstrate that for alphaviruses belonging to the SFV complex, the nsP4 replicase component appears to determine the recognition/efficient use of the SG promoter. For the genomic promoter, the similar correlation could not always be observed, either due to specific properties of replicase components belonging to different viruses or due to lower sensitivity of the corresponding assay.

## DISCUSSION

Significant progress toward understanding the RNA replication process of alphaviruses has been made in recent years. Three-dimensional structures of nsP1, nsP2, and the N-terminal two-thirds of nsP3 have become available (22, 58, 59) together with information about the significance of primary and higher-order RNA structures located in the terminal regions of the alphavirus genome (41, 60, 61). Progress has been made in understanding the formation of alphavirus RC structures and their functioning (21, 45, 49, 62), as well as in revealing the host factors that are required for alphavirus RNA replication (9, 10, 12). These developments have highlighted the need for studies aiming to understand how these essential components, structures, and processes work together (11, 14). In addition to basic information about the molecular biology of alphaviruses, these studies provide an improved understanding of the possible mechanisms of alphavirus evolution, including host and vector switches (17). Furthermore, they point to novel, promising technologies for virus attenuation and their use in bio- and gene technology applications to control arboviruses. Here, we extended these studies by providing novel insights about the compatibility of the main components of the alphavirus RNA replication machinery.

The nsP4 RNA-dependent RNA-polymerase is the most conserved protein between alphavirus species. Therefore, the finding that many heterologous combinations of P123 and nsP4 components of viruses belonging to the SFV complex resulted in a functional RNA replicase is not surprising. Interestingly, with few exceptions, the P123 components of these viruses formed active replicases also with the nsP4 proteins of outgroup viruses, including EILV, which is an ISV (Fig. 4). However, the strong asymmetry observed in the formation of a functional RNA replicase was unexpected. nsP4 proteins of SINV and VEEV can form functional replicases with P123 components of all members of the SFV complex and BFV, but the P123 components of VEEV and SINV were virtually unable to form an active replicase with any heterologous nsP4 protein (Fig. 5C to E), and nsP4 of BFV was functionally incompatible with any heterologous P123 (Fig. 4 and 5). Although less pronounced, the elements of such asymmetry were also observed for viruses belonging to the SFV complex. For example, P123 of RRV was able to form an active replicase with the nsP4 protein of all viruses belonging to the SFV complex (Fig. 4D), while nsP4 of RRV, when combined with P123 of ONNV, MAYV, or SFV displayed almost no transcription activity (Fig. 4). The reasons for this incompatibility likely vary between different viruses. Thus, for combinations involving the nsP4 or P123 components of EILV replicase, the incompatibility may originate from insect-cell specificity of EILV and/or reflect the finding that in order to form an active RNA replicase in mammalian cells, plasmid expressing EILV P123 should be combined with an excessive amount of nsP4 expression plasmid (Fig. 3). For viruses belonging to the SFV complex, such as SFV and RRV, the incompatibility may be caused by some specific properties of the *trans-*replicase system, as in the context of a chimeric genome, the combination of SFV P123 and RRV nsP4 resulted in viable virus, albeit with reduced capacity for CPE development and capsid protein expression (Fig. 6, Table 2). The impact of incompatibility of the replicase components was clearly more severe in the case of SINV, where the chimeric viruses containing heterologous nsP4 proteins were not viable at 37°C. Presumably, this may also be the case for similar VEEV chimeras, but due to biosafety concerns, we were unable to verify this experimentally. Somewhat surprisingly, all SINV chimeras were viable at 28°C. We have previously observed that some mutant alphaviruses are unable to grow at 37°C but can do so at reduced temperatures (57, 63). For classical temperature-sensitive mutations, such an effect is mostly caused by misfolding of mutant proteins at elevated temperatures. As all SINV chimeras used in this study expressed only ns-proteins from WT viruses, this was unlikely to be the case here. While it cannot be excluded that at 37°C SINV P123 failed to support correct folding of heterologous nsP4 proteins, it is more likely that the reduced temperature allowed sufficiently strong and stable interaction between suboptimally compatible components of the RNA replicase.

The cross-utilization of template RNAs by alphavirus replicases in mammalian and mosquito cells is similar (11). Currently, it is not known if the same compatibility also applies to the P123 and nsP4 replicase components of different viruses. If this indeed is the case, it may indicate that mosquitoes are the most likely places for interspecies recombination of alphaviruses. Viable hybrids of ISVs and arboviruses can be theoretically formed due to the ability of their replicases to cross-utilize template RNAs (11). Here, for the first time, we also demonstrate that the replicase components of these viruses can be swapped as well. Furthermore, the nsP4 of EILV is fully capable of functioning in mammalian cells and, albeit with somewhat reduced efficiency, also at temperatures characteristic for mammals. Thus, our data show that genomic recombination between ISV and an arbovirus presents another possible evolutionary trajectory that may lead to the emergence of new alphavirus species.

The compatibility of P123 and nsP4 components of different alphaviruses can be used for the development of viable but attenuated viruses that may serve as vaccine candidates. The genomes of such viruses would contain hundreds of nucleotide differences compared to either parental virus, making reversions virtually impossible. This leaves the use of adaptive mutations as the only option to increase their replication efficiency, but at least in our experience, mutant alphaviruses that have acquired adaptive changes always remain attenuated. Notably, it is possible that the rescue of chimeric viruses based on SFV and SINV genomes (Fig. 6) also involved the acquisition of adaptive mutations. Theoretically, the localization and nature of such mutations could provide important information about the interaction of different components of RNA replicases. However, our parallel study analyzing chimeric SFV genomes where domains of nsP2 or nsP3 were swapped with homologous domains of CHIKV and SINV revealed that this is generally not the case. Numerous adaptive mutations were indeed detected in the genomes of rescued viruses, but most of these mutations acted via a general increase of replication efficiency by altering the ns-polyprotein processing, through the increase of RNA polymerase activity of nsP4, and/or by altering the sequence of the SG promoter (see the companion article [16]). For this reason, analysis of potential adaptive mutations in the SFV and SINV chimeras was not performed in this study.

Several lines of evidence point to nsP4 as a key factor for the efficiency of alphavirus RNA synthesis. First, increasing the relative amount of nsP4 strikingly increased the RNA synthesis for all analyzed two-component replicases (Fig. 3A). A similar observation has been previously made using ONNV and CHIKV mutants that lack an opal termination codon between the nsP3 and nsP4 regions (64, 65). Thus, the effect observed in the *trans*-replication system accurately reflects the situation with infectious viruses, though possibly in a more pronounced manner. It is not known how the excess of nsP4 increases the efficiency of RNA synthesis. One of the possible explanations is that an excessive amount of nsP4 could be incorporated into RCs, making them more active. Alternatively—and more likely—an excess of nsP4 allows the formation of a larger number of RCs per infected/transfected cell. To distinguish between these possibilities, the numbers of RCs in cells transfected using different ratios of nsP4 and P123-encoding plasmids should be compared. Unfortunately, correlative light-electron microscopy, required for these experiments, is poorly suited for quantification. Another important question is why alphaviruses suppress their nsP4 expression by having a leaky stop codon at the end of the nsP3-encoding region and allowing rapid degradation of nsP4 by the N-end rule pathway (54). The likely answer to this question is that while an excess of nsP4 increases virus RNA synthesis and can enhance virus multiplication in cell culture (65), it is also associated with prominent negative consequences. First, we have shown that the proteasome inhibitor bortezomib increases the amount of nsP4 (and other ns-proteins) in CHIKV-infected cells, but nevertheless, the compound acts as an inhibitor of CHIKV replication (66). Thus, the excessive amounts of nsP4 have a negative impact on other key steps of the alphavirus infection process. This may also be a reason for the duplicated mechanism used to control the levels of nsP4 in infected cells. Thus, it has been shown that the replacement of the opal stop codon in the nsP3 region of CHIKV with an Arg codon results in an increased translation of the downstream region but does not necessarily result in a detectable increase in levels of mature nsP4 in infected cells; however, the opal to arginine substitution did enhance viral fitness when it was combined with two mutations in nsP1 (67). The negative effects caused by the excess of nsP4 may also include a disturbance in the processes needed to complete the virus replication cycle (66). It has also been shown that the excess of nsP4 due to the replacement of the opal termination codon with an argine codon altered CHIKV-induced pathology since the mutant virus caused significantly less swelling, inflammation, and damage in the feet and ankles of infected mice (65).

Our analysis revealed that for viruses belonging to the SFV complex, nsP4 is most likely the replicase component responsible for the selectivity of the SG promoter; data also indicate that it is probably responsible for selectivity of the genomic promoter as well (Fig. 8). Given that nsP4 is highly conserved and, for SINV, interacts specifically with sequences of the genomic and SG promoters (68, 69), this may apply to outgroup alphaviruses as well. If so, there is an analogy with NS5 RNA polymerase of flaviviruses, which has been demonstrated to recognize RNA structures inside the 5′ untranslated region (UTR) of these viruses and bind to these RNA elements with a 1:1 stoichiometry (70). Unfortunately, obtaining similar data for alphavirus nsP4 proteins will be challenging due to the poor solubility of recombinant nsP4 proteins. An active recombinant full-length nsP4 of CHIKV could not be obtained using bacterial expression systems, and even the core part of the protein, lacking the disordered N-terminal region, had very poor solubility and very low activities (30). For the same reason, nsP4 is the only alphavirus ns-protein for which the three-dimensional structure is not yet available, precluding the use of molecular modeling for detection of possible protein:RNA interactions. Interestingly, the solubility of the recombinant protein corresponding to the core region of nsP4 of SINV is higher, and the protein has strong terminal adenylyltransferase activity (71). This was also demonstrated for the RNA polymerase activity of the recombinant full-length nsP4 of SINV (29). Here, we have shown that the nsP4 of SINV or other alphaviruses can functionally substitute the nsP4 of CHIKV (Fig. 4A and Fig. 6). This finding indicates that structural studies performed using nsP4 proteins that have better solubility/activity than nsP4 of CHIKV would likely provide relevant information regarding the structure and functions of CHIKV nsP4 and possibly also provide insight on alphavirus nsP4/RNA interactions in general.

It was also observed that several biological properties of alphavirus replicases are associated with its P123 component. Thus, the previously observed poor activity of ONNV *trans-*replicase is due to specific properties of the P123 component. In contrast, the nsP4 component of ONNV replicase was highly active when combined with heterologous P123 components (Fig. 4 and 5). Identifying which part(s) of the P123 region of ONNV is responsible for the observed phenotype and how this reflects on *in vitro* and *in vivo* replication of the virus represent topics of ongoing studies. It was also observed that splitting the P12^CA^3 component into nsP1 and P2^CA^3 does have a different impact for the replicases of different viruses—milder for SINV while more pronounced for CHIKV RNA synthesis (Fig. 7). As of now, the extent of reduction of RNA synthesis seems to be related to the individual virus rather than reflect a common property of viruses belonging to the SFV complex; it has been reported that modified (green-fluorescent protein [GFP] inserted in the nsP3 region) nsP1+P2^CA^3+nsP4 replicase of SFV has an activity similar to that of the corresponding P12^CA^3+nsP4 replicase (49), while our attempts to construct an active three-component replicase of RRV were not successful. It is also interesting that, unlike for two-component replicases, the performance of the three-component replicases could not be substantially improved by altering the ratios of the plasmids encoding nsP1, P2^CA^3, and nsP4 components. This may reflect the different nature of the interaction between P123 and nsP4 compared to interactions of nsP1 with P2^CA^3 and/or nsP4. It is also possible that such interactions are mediated by additional components of the RC, such as template RNA and/or host factors. Detailed analysis of the interactions between the components of the RC, combined with information about protein structures, such as the recently revealed three-dimensional structure of nsP1 (22), is needed for understanding the molecular bases of this phenomenon as well as for better understanding of the principles of alphavirus RC formation in general.

## MATERIALS AND METHODS

### Cells and viruses.

HEK293T human embryonic kidney cells (ATCC CRL-3216) were maintained in Dulbecco’s modified Eagle medium (DMEM) with 2 mM l-glutamine and 10% fetal bovine serum (FBS) at 37°C in a 5% CO_2_ atmosphere. U2OS human bone osteosarcoma cells (ATCC HTB-96) were maintained in Iscove’s modified Dulbecco’s medium (Gibco) containing 10% FBS and 2 mM l-glutamine at 37°C in a 5% CO_2_ atmosphere. BHK-21 baby hamster kidney cells (ATCC CCL-10) were grown in Glasgow’s minimal essential medium (Gibco) containing 10% FBS, 2% tryptose phosphate broth (TPB), and 200 mM HEPES, pH 7.2, at 37°C in a 5% CO_2_ atmosphere. All media were supplemented with 100 U/ml penicillin and 0.1 mg/ml streptomycin.

### Plasmids.

Plasmids encoding P1234 of selected alphavirus and designated CMV-P1234-CHIKV, CMV-P1234-ONNV, CMV-P1234-RRV, CMV-P1234-SFV, CMV-P1234-MAYV, CMV-P1234-SINV, CMV-P1234-BFV, CMV-P1234-VEEV, and CMV-P1234-EILV, as well as their variants encoding polymerase-negative variant P1234^GAA^ (CMV-P1234^GAA^-CHIKV and so on) have been previously described together with human RNA polymerase I promoter-based plasmids for the production of replication-competent RNA templates (HSPolI-FZsG-CHIKV, HSPolI-FG-CHIKV, and so on) (11, 13).

Plasmids for expression of P123 of selected alphaviruses were constructed using PCR-amplified fragments and P1234 polyprotein expression plasmids; the obtained constructs were designated CMV-P123-CHIKV and so on. A similar approach was used to obtain plasmids expressing nsP1 of CHIKV, RRV, and SINV designated CMV-nsP1-CHIKV, CMV-nsP1-RRV, and CMV-nsP1-SINV. Plasmids expressing nsP4 fused to ubiquitin at their N termini were constructed using synthetic DNAs (Genscript) and fragments of P1234 expression plasmids; the resulting constructs were designated CMV-ubi-nsP4-CHIKV and so on. In plasmids CMV-P12^CA^3-CHIKV, CMV-P12^CA^3-RRV, and CMV-P12^CA^3-SINV, codons corresponding to the catalytic Cys residue in the active site of nsP2 protease (residue 478 for CHIKV and RRV and residue 481 for SINV) were replaced with codons for Ala using PCR-based mutagenesis and subcloning procedures. In plasmids CMV-ubiP2^CA^3-CHIKV, CMV-ubiP2^CA^3-RRV, and CMV-uniP2^CA^3-SINV, the nsP1-encoding region of CMV-P12^CA^3-CHIKV, CMV-P12^CA^3-RRV, and CMV-P12^CA^3-SINV was replaced by a region encoding ubiquitin.

The icDNAs of SINV with their nsP4 region swapped for CHIKV, ONNV, BFV, RRV, SFV, MAYV, VEEV, or EILV were constructed using icDNA plasmid pToto1101 (72) and synthetic DNA fragments (Genscript). In each of these fragments, synonymous mutations were introduced into the regions encoding 40 C-terminal amino acid residues of nsP4 (Table 1). When cloned into pToto1101, the insert replaced the nsP4 region of SINV while preserving the natural SG promoter region from position −98 with respect to the SG RNA transcription start site; viruses corresponding to these icDNAs were designated SINV-CHIKV4, SINV-ONNV4, SINV-BFV4, SINV-RRV4, SINV-SFV4, SINV-MAYV4, SINV-VEEV4, and SINV-EILV4 (Fig. 6A). In order to account for the potential impact originating from introduced modifications, an icDNA corresponding to a virus designated SINV-SINV4 containing a modified nsP4 region of SINV (Table 1) was constructed using the same approach. icDNA clones of SFV with their nsP4 region originating from CHIKV, BFV, RRV, or SINV were constructed using icDNA plasmid pCMV-SFV6 (56) and synthetic DNA fragments as described for icDNAs of SINV. The viruses obtained from these icDNA clones were designated SFV-CHIKV4, SFV-BFV4, SFV-RRV4, and SFV-SINV4; the control virus constructed using nsP4 encoding fragment of SFV was designated SFV-SFV4 (Fig. 6A).

All DNA manipulations were performed using restriction enzyme-based cloning methods. The sequences of all plasmids were verified using Sanger sequencing and are available from the authors upon request.

### Rescue of recombinant viruses.

Virus rescue and ICA were performed as previously described (73, 74). Briefly, in order to rescue SINV and corresponding chimeric viruses, BHK-21 cells (8 × 10^6^) were transfected with capped *in vitro* transcripts from icDNA clones by electroporation with a Bio-Rad Gene Pulser II unit (two pulses at 850 V and 25 μF) in 0.4-cm cuvettes (Thermo Fisher Scientific). In order to rescue SFV and corresponding chimeric viruses, 5 μg of endotoxin-free plasmids corresponding to SFV6, SFV-SFV4, SFV-CHIKV4, SFV-BFV4, SFV-RRV4, and SFV-SINV4 were used for transfection of BHK-21 cells (one pulse at 220 V and 975 μF).

Then, 10-fold dilutions of cells were prepared using 10% transfected cells and seeded onto 6-well tissue culture plates containing 1.5 × 10^6^ BHK-21 cells per well. After 2 h of incubation at 37°C, the cell culture medium was aspirated, and the cells were overlaid with 2 ml of growth medium supplemented with 0.8% carboxymethyl cellulose (Sigma Life Science). Plaques were stained with crystal violet after 2 to 3 days of incubation at 37°C and counted, and the infectivity in PFU per μg of RNA or plasmid DNA was calculated.

The remaining of transfected cells were divided in half. The first half was plated into a 6-well plate and incubated at 37°C, and viral stocks were harvested at 24 h posttransfection (p.t.) or 72 h p.t. (Table 2). The other half of the transfected cells was plated as described above, but plates were incubated at 28°C and virus stocks were collected at 48 to 96 h p.t. (Table 2). The obtained stocks were clarified by centrifugation at 3,000 × *g* for 10 min. Then, 10% of the collected virus stock was used to infect fresh BHK-21 cells. The cells were incubated at 37°C or 28°C until CPE was detected or for up to 72 h (Table 2). All assays were repeated twice.

### Western blotting.

Cells transfected using icDNA plasmids (SFV) or *in vitro* transcripts (SINV) corresponding to WT and chimeric viruses were harvested at the same time as the corresponding viral stocks. After washing with 1 ml of PBS, cells were lysed and boiled, and proteins were separated using SDS-PAGE in 10% gels. The separated proteins were transferred to polyvinylidene difluoride membranes and detected using antibodies against SFV nsP2, SFV capsid protein, SINV nsP2 (all in-house), SINV capsid protein (a kind gift from Diane E. Griffin, Johns Hopkins University, USA) and, β-actin (sc-47778; Santa Cruz Biotechnology). After incubating with appropriate secondary antibodies conjugated to fluorescent labels (LI-COR), the proteins on the membrane were imaged using the LI-COR Odyssey Fc imaging system.

### *Trans*-replication assay.

For HEK293T, the *trans*-replication assay was carried out using a 96-well-plate format as previously described (57). Briefly, approximately 35,000 cells per well were cotransfected with 50 ng of plasmids encoding template RNA and 50 ng of plasmids encoding P1234. For two-component replicases, 50 ng of P123 (or P12^CA^3)-encoding plasmid was combined with an equimolar amount of nsP4 expression plasmid. In order to determine the dependence of RNA synthesis efficiency from the ratio of nsP4 to P123 (or P12^CA^3), molar ratios of nsP4 and P123 (or P12^CA^3)-encoding plasmids of 1:10, 2:10, 4:10, 6:1, 8:10, 1:1, 2:1, 4:1, 6:1, and 8:1 were used. As negative controls, CMV-P1234^GAA^ was used instead of CMV-P123 (or CMV-P12^CA^3) and CMV-ubi-nsP4. Transfections were performed using FuGENE 6 reagent (Promega) according to the manufacturer’s instructions. Transfected cells were incubated at 37°C for 18 h; as an exception, the cells transfected with EILV P123 and nsP4 expression plasmids were incubated at 28°C for 48 h.

U2OS cells grown on 12-well plates were cotransfected with 1 μg of HSPolI-FG-CHIKV or HSPolI-FG-SINV and 1 μg of CMV-P1234-CHIKV or CMV-P1234-SINV using Lipofectamine LTX with PLUS reagent (Thermo Fisher Scientific) according to the manufacturer’s instructions; in control cells, the latter was replaced with plasmid encoding a polymerase-negative version of replicase protein (CMV-P1234^GAA^-CHIKV and CMV-P1234^GAA^-SINV). For two-component replicases, CMV-P1234-CHIKV or CMV-P1234-SINV was substituted with 1 μg of P123 (or P12^CA^3)-encoding plasmid that was combined with an equimolar amount of nsP4 expression plasmid; for three-component replicases, 1 μg of P2^CA^3-encoding plasmid was combined with equimolar amounts of nsP1 and nsP4 expression plasmids. Transfected cells were incubated at 37°C for 18 h.

All transfections were performed in triplicate, and experiments were repeated at least twice. After incubation, cells were lysed, and Fluc and Gluc activities were measured using the dual-Luciferase-reporter assay (Promega). Fluc and Gluc activities measured for cells transfected using plasmids expressing active replicases were normalized to those obtained for the corresponding control cells.

### Flow cytometry assay.

Approximately 35,000 HEK293T cells grown in 96-well plates were cotransfected with 50 ng of HSPolI-FZsG-CHIKV and 50 ng of CMV-CHIKV-P123 or CMV-CHIKV-P12^CA^3 combined with CMV-ubi-nsP4-CHIKV using the following molar ratios of nsP4- and P123 (or P12^CA^3)-encoding plasmids: 1:10, 2:10, 4:10, 6:1, 8:10, 1:1, 2:1, 4:1, 6:1, and 8:1. As a negative control, CMV-P1234^GAA^-CHIKV was used instead of P123 (or P12^CA^3) and nsP4 expression plasmids. Transfections were performed using Lipofectamine LTX with PLUS reagent (Thermo Fisher Scientific) according to the manufacturer’s instructions. Transfected cells were incubated and analyzed as described above except that only Fluc activity was measured.

Approximately 400,000 HEK293T cells were grown in 24-well plates and cotransfected with 250 ng of HSPolI-FZsG-CHIKV, 250 ng of CMV-P123-CHIKV, or CMV-P12^CA^3-CHIKV combined with CMV-ubi-nsP4-CHIKV using the following molar ratios of nsP4- and P123 (or P12^CA^3)-encoding plasmids: 1:10, 2:10, 4:10, 6:1, 8:10, 1:1, 2:1, 4:1, 6:1, and 8:1. Transfections were performed using Lipofectamine LTX with PLUS reagent. Cells were collected in 500 μl PBS 18 h p.t. and analyzed with an Attune NxT acoustic focusing cytometer. At least 30,000 events were recorded for each sample. The obtained data were analyzed using Attune NxT software to determine the percentage of living cells, the percentage of cells positive for ZsGreen marker, and the mean fluorescence intensity of ZsGreen (in arbitrary units) in marker-positive cells. The experiment was repeated three times.

### Northern blotting.

Northern blotting was performed as previously described (11). Briefly, HEK293T cells grown in 12-well plates were cotransfected with 1 μg of template RNA expression plasmid and 1 μg of P123 expression plasmid combined with an equimolar amount of nsP4 expression plasmid using Lipofectamine LTX with PLUS reagent; control cells were mock-transfected. At 18 h p.t., total RNA was extracted using TRIzol reagent (Life Technologies). Then, 2 μg of total RNA was used for detection of positive strands, and 10 μg of total RNA was used for detection of negative strands. RNAs were denatured for 10 min at 70°C in 2× RNA loading dye (Thermo Fisher Scientific), cooled on ice, and separated on a denaturing gel (1% agarose/6% formaldehyde) using 1× MOPS (morpholinepropanesulfonic acid) buffer. RNA was transferred to a Hybond-N+ filter (GE Healthcare) and fixed using a UV Stratalinker 1800 (Stratagene). Digoxigenin (DIG)-labeled RNA probe complementary to residues 42 to 390 of the sequence encoding Gluc marker was used to detect positive-strand RNAs; probe corresponding to residues 51 to 376 of the sequence encoding Fluc marker was used to detect negative-strand RNAs. Filters were hybridized overnight; blots were washed and developed according to the manufacturer’s (Roche) protocols.

### Statistical analysis.

Statistical analysis was performed using GraphPad Prism 8.2.0 software. Data were analyzed using Student’s unpaired one-tailed *t* test. *P* values of ≤0.05 (*), ≤0.01 (**), ≤0.001 (***), and ≤0.0001 (****) were used to represent degrees of significance. Each experiment was repeated to gain a minimum of 3 independent biological repeats.
